# The branched-chain amino acid transporter AAT10.2 of *Trypanosoma brucei* is regulated by substrate availability

**DOI:** 10.1016/j.jbc.2026.113379

**Published:** 2026-07-28

**Authors:** Quentin-Florian Oliveres, Alexander C. Haindrich, Silvan Piller, Juan P. Macedo, Veerle van Berkum, Fabio Luiz Rogé Ferreira, Christoph Wenger, Arunasalam Naguleswaran, Doris Rentsch

**Affiliations:** 1 Institute of Plant Sciences, University of Bern, Bern, Switzerland; 2 Molecular Plant Physiology and Biophysics, Julius-von-Sachs-Institute, Julius-Maximilians-Universität Würzburg, Wuerzburg, Germany; 3 Institute of Animal Pathology, University of Bern, Switzerland

**Keywords:** amino acid, transport, parasite, phosphoproteomics, substrate specificity, branched-chain amino acids

## Abstract

Amino acid transporters (AATs) are essential for the uptake of exogenous amino acids, and their regulation allows the protozoan parasite *Trypanosoma brucei* to rapidly adapt to changing environmental conditions when alternating between the mammalian and the insect host, or when invading different host tissues. With a phosphoproteome analysis of procyclic form *T. brucei* we identified many AATs of the amino acid/auxin permease (AAAP) family with phosphorylated residues exclusively in their hydrophilic N-terminal domain. AAT10.2, which contained multiple phosphorylated residues, was further characterized and shown to be localized at the plasma membrane and the flagellar pocket. RNAi in procyclic form *T. brucei* revealed that AAT10.2 is essential and the main transporter for branched-chain amino acids (BCAA) under the conditions tested. High affinity and selective transport of BCAA was verified by expressing AAT10.2 in *Saccharomyces cerevisiae*. In *T. brucei*, AAT10.2 protein abundance was regulated by BCAA levels. However, mutation of the identified phosphorylation sites or truncation of the first 35 amino acids did not affect affinity, localization, or the BCAA-dependent regulation of AAT10.2. The non-phosphorylatable 3xTy-STA-AAT10.2∗ variant, in which N-terminal serine and threonine residues were mutated to alanine, showed increased protein levels compared to native Ty-tagged AAT10.2∗ or the phosphomimicking variant (3xTy-STD-AAT10.2∗) with aspartate replacing serine and threonine in the N-terminal region. Truncation of the N-terminal domain reduced the overall AAT10.2 abundance and BCAA levels in short-term experiments, and decreased *T. brucei* growth when BCAA availability was limited. These findings establish AAT10.2 as an essential, substrate-regulated BCAA transporter in procyclic form *T. brucei*.

*Trypanosoma brucei* is a protozoan parasite and the causative agent of sleeping sickness in humans and nagana in animals. Transmitted by infected tsetse flies (*Glossina* genus), human African trypanosomiasis primarily affects people in remote rural areas. Additionally, *T. brucei* trypanosomiasis in domestic animals poses a significant economic burden that is difficult to estimate (1, 2). *T. brucei* has a complex life cycle, thereby alternating between mammalian and insect hosts, which is associated with developmental and morphological changes. Surviving in diverse environments also requires significant metabolic adaptation. In the mammalian host, the parasite must escape the hostile immune system, while in the tsetse fly midgut it encounters a proteolytic environment and varying nutrient availability (3, 4, 5, 6).

Like other trypanosomatids, *T. brucei* is auxotrophic for many amino acids, including the branched-chain amino acids (BCAA) leucine, isoleucine, and valine (7, 8). In addition to their role in protein synthesis, BCAA can contribute to various metabolic pathways. BCAA degradation starts with deamination, and thus BCAA may serve as nitrogen donors (9). Leucine catabolism was studied in the related parasites *Leishmania* (8, 10) and *Trypanosoma cruzi* (8, 11). In these parasites, leucine was described to finally be metabolized through the TCA cycle and serve as a precursor for both fatty acid and sterol biosynthesis (12, 13, 14). In *T. brucei* not all genes required for the complete degradation of BCAA were identified (15), and whether other genes may take over the missing functions is currently unclear. In contrast to *T. cruzi* and *Leishmania*, none of the BCAA was found to be used for fatty acid synthesis in *T. brucei* (16). Leucine and, to a reduced extent, also isoleucine were reported to participate in *de novo* sterol biosynthesis, whereas valine was not utilized as a precursor (16). However, leucine is not the preferred carbon source for sterol synthesis and at least under culture conditions, both bloodstream form (BSF) and procyclic form (PCF) *T. brucei*, prevalent in the human blood or the insect midgut, respectively, prefer using acetate derived from threonine and glucose (16). Notably, PCF *T. brucei* can compensate for a decreased availability of these preferred sterol precursors by increasing the incorporation of leucine (16).

In general, the loss of *de novo* biosynthesis pathways reflects a response to nutrient availability, a consequence of a heterotrophic or parasitic lifestyle (7). A loss of different biosynthetic pathways was already observed in a free-living, phagotrophic ancestral trypanosomatid, while multiplication of transporters, *e*.*g*. for amino acids or nucleosides, seems special for parasitic trypanosomatids (17). Therefore, in *T*. *brucei*, transporters are predicted to play a crucial role in parasite metabolism and survival.

*T. brucei* possesses a particularly wide expansion of its amino acid transporter repertoire, and by gene duplications and mutations acquired a total of 47 predicted amino acid transporter (AAT) genes (18). Most AATs belong to the amino acid/auxin permease (AAAP) family, a widespread family of secondary active transporters with members in humans, plants, and fungi, while three genes annotated as AATs are members of the closely related amino acid-polyamine-organocation (APC) transporter family. AAAPs share a LeuT-fold and have generally 11 predicted transmembrane domains, with the hydrophilic N-terminal region predicted to be oriented towards the cytosol (19, 20, 21, 22). Transport is typically energized by the co-transport of proton or sodium ions (23). The majority of *T. brucei* AATs show high sequence similarity among each other. However, the number of suitable amino acids in transmembrane domains is restricted by the hydrophobic environment, and therefore, the hydrophilic N-terminal domains as well as internal loops correspond to the most variable regions.

To date, only a few of these AATs have been studied in detail. The first functionally characterised transporter was AAT6, which was initially identified as the entry gate for the drug eflornithine (24, 25, 26), and was later shown to be a low-selective transporter for various neutral amino acids (27). AAT10.1 and AAT2.4 were reported to transport ornithine or ornithine and histidine, respectively, and to maintain intracellular ornithine and polyamine levels in BSF parasites. Lower ornithine uptake increased eflornithine sensitivity and decreased sensitivity to another drug, *i*.*e*. suramin (28) and therefore linked these transporters to drug sensitivity. Further characterized *T. brucei* AATs include the high-affinity transporters for arginine (AAT5.1 to AAT5.5) and lysine (AAT16.1 and AAT16.2) that are essential in BSF *T. brucei* (29), and the three members of the proline/alanine transporter clade (AAT7-B) (30), which are upregulated during amino acid or glucose starvation, and essential in PCF *T. brucei* under limiting substrate availability.

In agreement with a role of amino acid transporters in adaptation to changing nutrient availability, transcript levels of AATs were not only reported to be differentially regulated during the life cycle of *T. brucei* (5), but were also shown to be dependent on glucose availability (31). Furthermore, Haindrich *et al*. (30) identified four AAT genes among the ten most upregulated transcripts when PCF parasites were exposed to low amino acid availability.

During the transmission of *T. brucei* from one host to another, rapid changes in osmolarity, temperature, pH and nutrient composition occur, and the parasites are challenged by host-specific defense mechanisms. Therefore, parasites require fast adaptation skills. In addition to rapid adjustments of transcript levels, post-translational modifications represent a quick and versatile mechanism to regulate protein activity. For example, in *Leishmania*, arginine transporter LdAAP3 mRNA and transport activity were shown to be regulated by external arginine levels through a protein kinase-dependent arginine deprivation response pathway (32, 33). Phosphorylation, a post-translational protein modification, reversibly modifies proteins by attachment of a phosphate group on serine, tyrosine, or threonine residues. In *T. brucei*, reversible phosphorylation is a well-established mechanism shown to regulate, for example, protein activity (34), localization (35), and to control protein–protein interactions (36). A role of phosphorylation of transporters has been shown in many different organisms and was demonstrated to modify transport activity (37, 38), affinity (37, 39), membrane trafficking (37, 40, 41) and protein turnover (42); however, the role of phosphorylation in the regulation of transporter proteins in *T. brucei* remains largely unexplored.

AAT10.2 (Tb927.8.8300), encoded by the second gene of the AAT10 locus, was repeatedly found in phosphoproteome analyses (43, 44, 45, 46), and AAT10.2 protein levels were reported to be 10- to 14-fold higher in PCF *T. brucei* than in BSF parasites (45, 46). Additionally, individual phosphorylation sites in the hydrophilic N-terminal region were approximately 3- to 13-times more phosphorylated in PCF than in BSF parasites (46). AAT10.2 shows high similarity (65% identity on amino acid level) to both AAT10.1 and AAT2.4, but in our previous study no ornithine or histidine transport activity could be shown for AAT10.2 when expressed in a heterologous system (28). Furthermore, when downregulated by RNAi, AAT10.2 was not found to be essential for BSF parasites under standard culture conditions (28).

In this study, we assessed the phosphoproteome of PCF *T. brucei* with a special focus on phosphorylation of AATs and confirmed that AAT10.2 is highly phosphorylated. Like in all other *T. brucei* AATs of the AAAP family, phosphorylation sites were found exclusively in the hydrophilic N-terminal domain. We further characterized the function of AAT10.2 as an essential BCAA transporter in PCF parasites and show AAT10.2 protein upregulation upon BCAA starvation.

## Results

To identify phosphorylation sites on AATs under our experimental growth conditions, enriched phosphopeptide fractions were isolated from PCF *T. brucei* (29-13) cultured in medium SDM79. We identified 1789 proteins with 4028 phosphorylation sites, of which 369 phosphoproteins were not previously described in PCF *T. brucei* (Fig. S1 and Data S1). Our analysis identified 15 to 24 phosphorylated AATs belonging to 12 AAT loci (Table 1). Due to the high sequence similarity of AAT proteins, the peptides identified are not in all cases unique; therefore, assignment of phosphopeptides to individual AATs is not always possible. AAT1, AAT9 and AAT17.2 had not been previously reported to be phosphorylated, though the probability for AAT17.2 was rather low (69%). In contrast, AAT10.1 and AAT14 were not among the phosphoproteins in our study (Table 1 and Data S1). Taken together, our data and published results (43, 46, 47) identified up to 26 phosphorylated AATs. In addition, there is some heterogeneity in the phosphorylated amino acid residues, *i*.*e*. some of the phosphorylation sites were previously found in one or several studies, whereas others were not reported before (Table 1).

**Table 1:** Phosphorylation sites of AATs

AAT locus	Protein IDs	Phosphorylated amino acid residues	Reference
AAT1	Tb927.4.3930	**T2;S10;S13**	Data S1
AAT3	Tb927.4.4730	*T19*;**T25;S37;S40;S44**;*S48*	Data S1
		S40;S44	(46)
		T25;S37;S40;S44	(43)
AAT4	Tb927.4.4820	**T20;S21;S25;S30**	Data S1
	Tb927.4.4840	S21;S25;S30	(46)
	Tb927.4.4860	S30	(43)
AAT5	Tb927.8.4740	**S3;S10**	Data S1
	Tb927.8.4720	S3	(47)
	Tb927.8.4700	S3;T5;S10	(43, 46)
	Tb11.v5.0569		
AAT6	Tb927.8.5450	*S70*;*S71*	Data S1
		T67;S70;S71	(43)
AAT7	Tb927.8.7700	*T31*;*S34*;**T57**	Data S1
	Tb927.8.7680	T57;S118	(46)
		S20	(43)
	Tb927.8.7670	*S11*;*T13*;*S21*;**S34**	Data S1
		S21;S34	(47)
		S34	(43, 46)
	Tb927.8.7610	*S11*;*T13*;**S21**	Data S1
		S8;S21;S34	(46)
		T6;S8;T10;S21;S34	(43)
AAT9	Tb927.8.8260	**S28**	Data S1
	Tb927.8.8250		
	Tb927.8.8230		
AAT10	Tb927.8.8290	S40;S43	(46)
	Tb927.8.8300	**S4;S6;S8;S11;S14;S19;T35**;*T40*;*S41*	Data S1
		S4;S6;T35;S40;S41	(47)
		S4;S6;S8;S11;S14;S19;T35	(46)
		S6;S27;T35;T40;S41	(43)
AAT12	Tb927.10.5780	*T50*;*S55*;**S58;S60;S65;S82;S85;T91**;*S102*;*S103*;**S146**	Data S1
		S36;S40;S46;S82;S85;T91	(47)
		S29;S46;S82;S85;T91;S107;S123;S146	(43)
AAT14	Tb927.10.9850	S3;S7	(43)
AAT15	Tb927.11.6680	**T500;S502**	Data S1
		S29;T41;T498;T500;S502	(46)
		S29;T500;S502	(43)
AAT16	Tb927.11.15860	*T17*;*S21*;*S23*;*S37*	Data S1
	Tb927.11.15840	S34;S37;T38	(46)
		S21;S23	(43)
AAT17	Tb927.11.15950	*S59*	Data S1
	Tb927.11.15960	S59	(43, 46)
		*T14*;*T15*;*S16*	Data S1

Phosphorylated residues identified in AATs (Table S2) and associated locus according to Jackson (18). Due to high sequence similarity, phosphorylated peptides may be assigned to multiple AATs. Phosphorylated residues identified in our study with a probability of localization ≥ 95% are shown in bold, 25 to 94% is shown in italics (n = 1).

With one exception, all phosphorylated residues of AATs are contained in the hydrophilic N-terminal domain (Table 1 and Data S1). This is in agreement with the predicted orientation and structure of members of the amino acid/auxin permease (AAAP) family, with 11 predicted transmembrane domains and the N-terminus facing the cytosol (19, 48). AAT15 (Tb927.11.6680) was the only transporter that was identified to be phosphorylated additionally near the C-terminus (Table 1). According to the transmembrane prediction tools DeepTMHMM or I-TASSER-MTD (49, 50), AAT15 contains 12 predicted transmembrane domains and therefore the C-terminal domain is predicted to be oriented towards the cytosol as well. In agreement with this topology is the phylogenetic relationship, *i*.*e*., although AAT15 (Tb927.11.6680) was classified as AAT (18), AAT15 is indeed a member of the polyamine transporter gene family and thus belongs to the amino acid-polyamine-organocation (APC) transporter gene family (and not the AAAP family). The APC and AAAP gene families belong to the same superfamily and are thus related, but there are also significant differences and, for example, transporters belonging to the APC have been shown to contain, in general 12 transmembrane domains (21).

Evidently, phosphorylation on hydrophilic, cytosolic end-domains is not a specific feature of AATs. Indeed, in our phosphoproteome analysis we found, for example the choline transporter Tb927.10.6730 or the putative transporter Tb927.10.14090 being phosphorylated on both their hydrophilic N- and C-terminal domains, while the glucose transporter Tb927.10.8480 was found to be phosphorylated only on its C-terminal domain. However, some transporters identified in our phosphoproteome analysis did not display these features, like for example the nucleobase (Tb11.v5.1052), purine (Tb927.9.7470), adenosine (Tb927.3.590), pteridine (Tb927.10.9080), or ABC (Tb927.11.6120) transporters, which were phosphorylated after their predicted sixth transmembrane domain or the folate transporter (Tb927.8.3650) that was phosphorylated on both the N-terminal domain and after the sixth predicted transmembrane domain (Data S1).

Among all AATs, phosphorylated AAT10.2 peptides were among the most abundant peptides in the phosphopeptide-enriched fraction (Tables 1, S1, and Data S1). Though our phosphoproteome data is not statistically verified (n = 1), a high number of phosphosites in AAT10.2 was also found in other studies (Table 1). The predicted hydrophilic N-terminal region of AAT10.2, *i*.*e*. amino acid 1 to 69, contained in total 10 phosphorylated serine and threonine residues (Table 1, (43, 46, 47)). The 9 sites identified in our analysis were present in different combinations (Table S2), suggesting that the overall charge rather than a specific phosphorylated residue is important. Additionally, AAT10.2 seems to be an abundant protein among AATs, based on the number of peptides found in the size-fractionated plasma membrane-enriched protein fraction analyzed by mass spectrometry (Table S1 and Data S2). In total the latter analysis identified up to 14 AATs belonging to 5 different loci (Table S1 and Data S2). These results and published data on phosphorylation of the N-terminus (43, 46, 47), together with reports on higher AAT10.2 protein abundance and higher phosphorylation in PCF than BSF parasites (45, 46), suggest a functional role of AAT10.2-phosphorylation in PCF *T*. *brucei*. However, as phosphorylated residues in N-terminal regions seem abundant also in other AATs, this modification may alternatively fulfill a more general role. Phosphorylation increases the negative charge, which may change the structure and accessibility of this domain.

### AAT10.2 is a BCAA transporter

**Figure 1:**
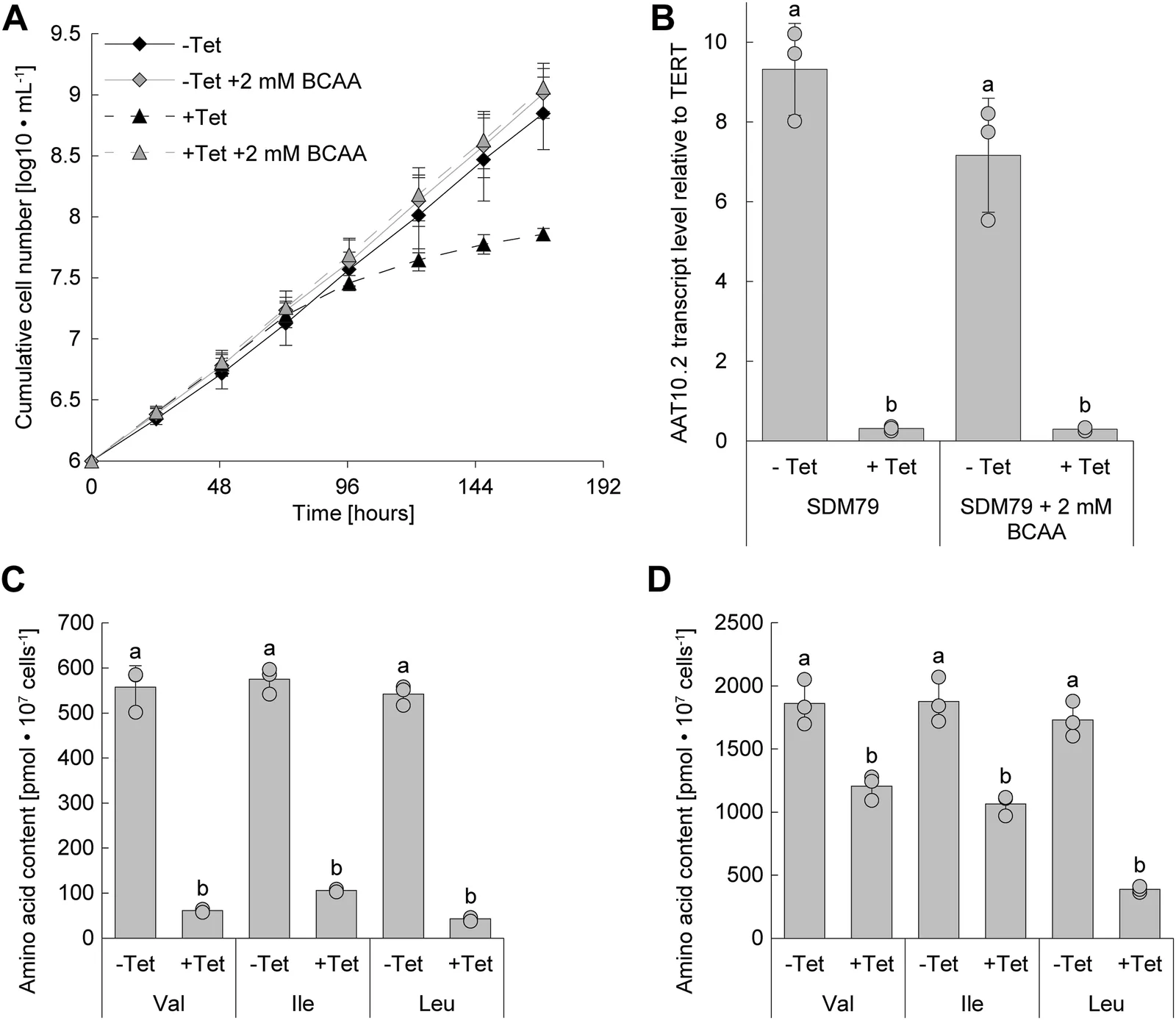
**Downregulation of AAT10.2 by RNAi in PCF *T. brucei* results in reduced growth and a decrease of valine, isoleucine, and leucine.***A*, cumulative growth of independent AAT10.2 RNAi lines (±Tet), supplemented or not with 2 mM BCAA and (*B*) associated AAT10.2 mRNA levels determined 72 h after RNAi induction and normalized to TERT. *C* and *D*, valine, isoleucine, and leucine content in AAT10.2 RNAi PCF *T. brucei* cells 72 h after induction of RNAi with tetracycline and in non-induced cells cultured in SDM79 (*C*) and SDM79 supplemented with 2 mM BCAA (*D*). Compact letters indicate statistically significant differences between treatment; error bar represent standard deviation (*p*-value<0.05, two-tailed *t* test, n = 3).

To investigate whether AAT10.2 is not only phylogenetically related to amino acid transporters, but indeed plays a role in amino acid uptake, AAT10.2 expression was downregulated by RNA interference (RNAi) in PCF *T. brucei* and intracellular amino acid levels were analyzed 24 h and 72 h after RNAi induction and compared to concentrations in non-induced cells (Table S3). While most amino acids remained unchanged or increased, only the level of BCAA was significantly reduced, and after 72 h of induction showed a decrease of valine, isoleucine and leucine by over 80% (Fig. 1*C* and Table S3*B*), suggesting that AAT10.2 is the main BCAA transporter in PCF *T. brucei*. Reduced cell growth could be rescued by the addition of 2 mM of each valine, isoleucine and leucine (Fig. 1*A*), however, all three BCAA were necessary to reach growth rates comparable to non-induced cells (Fig. S2). Concomitantly with growth rescue at 2 mM supplemented BCAA, also intracellular levels of BCAA were substantially elevated (Fig. 1*D* and Table S3*B*), but BCAA levels were still lower in induced AAT10.2 RNAi *T. brucei*. These results also showed that—at least in the presence of high BCAA—BCAA transport is not completely abolished in the induced AAT10.2 RNAi cells. qRT-PCR showed efficient downregulation of AAT10.2 transcript levels upon RNAi induction by tetracycline in the absence or presence of 2 mM BCAA (Fig. 1*B*).

**Figure 2:**
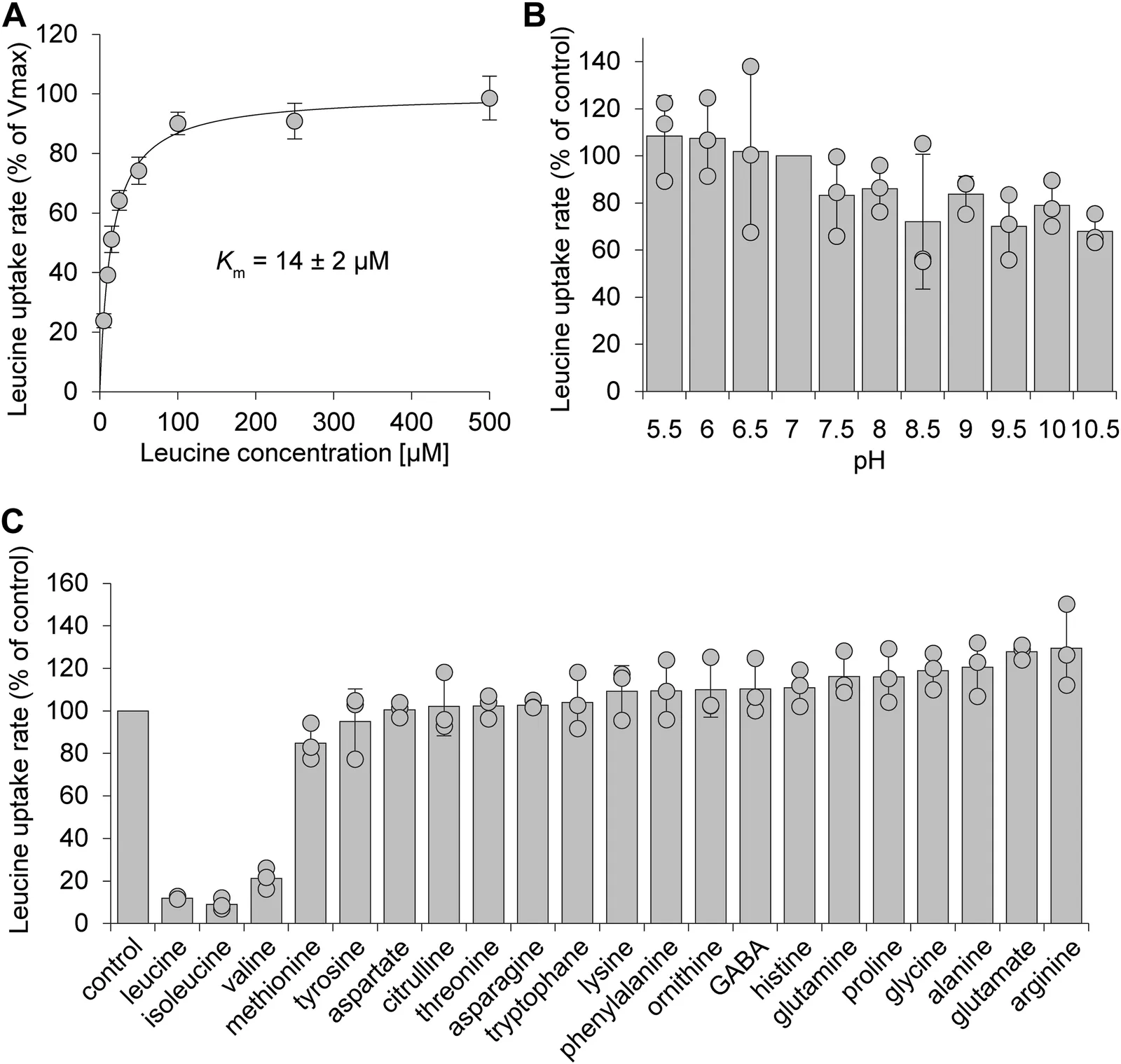
**AAT10.2 is a high-affinity leucine, valine and isoleucine transporter.** Uptake rates of L-[^3^H]-leucine were determined for AAT10.2 expressed in the *S. cerevisiae* mutant YDR544 as described in Experimental procedures. *A*, transport kinetics of AAT10.2 at pH 7.0. Apparent affinity was determined by using the Michaelis-Menten equation (n = 3, ± SD). Leucine transport rates at *V*_max_ were in the range of 30 to 100 pmol min^−1^ 10^6^ cells^−1^, which results in an internal concentration of approx. 1150 to 3820 μM (at 2.5 min). *B*, transport of 200 μM L-leucine at different pH values. Uptake rates of AAT10.2 expressed in *S. cerevisiae* were normalized to transport rates at pH 7 – set to 100% (n = 3, ± SD). *C*, uptake rates of 250 μM L-leucine in the presence of 2.5 mM competitors. Uptake rates are shown relative to the non-competed leucine transport rates-set to 100% (n = 3, ±SD). Dots show individual data points. GABA, γ-aminobutyric acid.

To confirm the role of AAT10.2 as a BCAA transporter, the open reading frame (ORF) was expressed in the *Saccharomyces cerevisiae* strain YDR544 that lacks several endogenous amino acid transporters (51). AAT10.2 supported time-dependent uptake of radiolabeled leucine, valine and isoleucine (Fig. S3). The apparent affinity of AAT10.2 for leucine was calculated by determining transport rates at different leucine concentrations at pH 7.0, comparable to the pH of *in vitro* cultured PCF *T. brucei* cells (pH 7.3). The apparent affinity was found to be 14 ± 2 μM (Fig. 2*A*). The [^3^H]-leucine accumulated in the cells exceeded the external concentration, suggesting active transport.

To identify other potential substrates of AAT10.2, competition experiments were performed by measuring leucine or isoleucine uptake rates in the presence of an excess of competitors. These transport studies showed that—with the exception of BCAA—other proteogenic amino acids, as well as ornithine, citrulline and γ-aminobutyric acid, showed no or only weak competition (Fig. 2*C*). Furthermore, the D-enantiomer of leucine did not compete for L-isoleucine uptake, indicating a potential role of the alpha carbon configuration in substrate recognition or transport (Fig. S4*B*). Other potential substrates structurally similar to BCAA or containing a leucine residue were also tested, such as the dipeptides Leu-Leu and Val-Leu, the tetrapeptide Leu-Ser-Lys-Leu (LSKL), norleucine, butyric acid, and butylamine, but none efficiently competed with isoleucine transport (Fig. S4*B*). These results thus supported the findings from the RNAi lines that AAT10.2 is a selective BCAA transporter.

Since *T. brucei* encounters various pH values throughout the different compartments of the tsetse fly (pH 7.6–10.6 (4, 52)), the uptake rates of AAT10.2 for leucine were measured at different pH values. The results showed that AAT10.2-supported leucine uptake remains functional over a wide pH range, from pH 5.5 to 10.5, with a tendency of increased transport activity at lower pH (Fig. 2*B*). As mentioned earlier, the accumulation of substrate against a concentration gradient argued for active transport. However, since the dependence of transport activity on pH is not pronounced, the question arose if AAT10.2-supported substrate transport is driven by proton symport, as is the case for many other members of the AAAP family (23). Alternatively, like for some human AAAP members, sodium ions could be the co-substrate (52). Na^+^ and H^+^ binding sites have been identified for several APC members (53, 54, 55), but Na^+^ binding is largely based on backbone carbonyl groups, and residues involved in H^+^ binding can be variable and their exact position is important, therefore, a homology-based identification without a crystal structure is difficult. An alignment of AAT10.2 with the recently crystalized AAAP transporter LAX3 of *Arabidopsis thaliana*, revealed that one histidine involved in proton binding, H319 in AtLAX3 is K350 in AAT10.2. A lysine residue was shown to be involved in proton binding in the APC transporter ApcT (54) and therefore K350 could have a similar function in AAT10.2. The second proton binding side formed by H247 in LAX3 could, however, not be identified in AAT10.2. When assessed in *S. cerevisiae*, changing the concentration of potassium or sodium ions did not influence the ability of AAT10.2 to facilitate leucine transport (Fig. S5). Expression in *Xenopus laevis* oocytes revealed inward currents, *i*.*e*. the inward movement of a positive charge, suggesting symport of the zwitterionic leucine, isoleucine, and valine with a cation (Fig. S6). Leucine-induced currents increased with decreasing membrane potential and elevated proton concentration, indicating co-transport of protons with an apparent affinity of 11 ± 5 μM (Fig. S6). However, the currents were too low to measure proton movement with a proton-sensitive electrode. Under the conditions tested (*i*.*e*. low pH gradient, resting potential of oocytes), AAT10.2 may also work in the reverse direction, as shown by higher efflux rates of radiolabeled leucine from oocytes expressing AAT10.2 than from control oocytes (Fig. S7).

### AAT10.2 localizes to the plasma membrane

**Figure 3:**
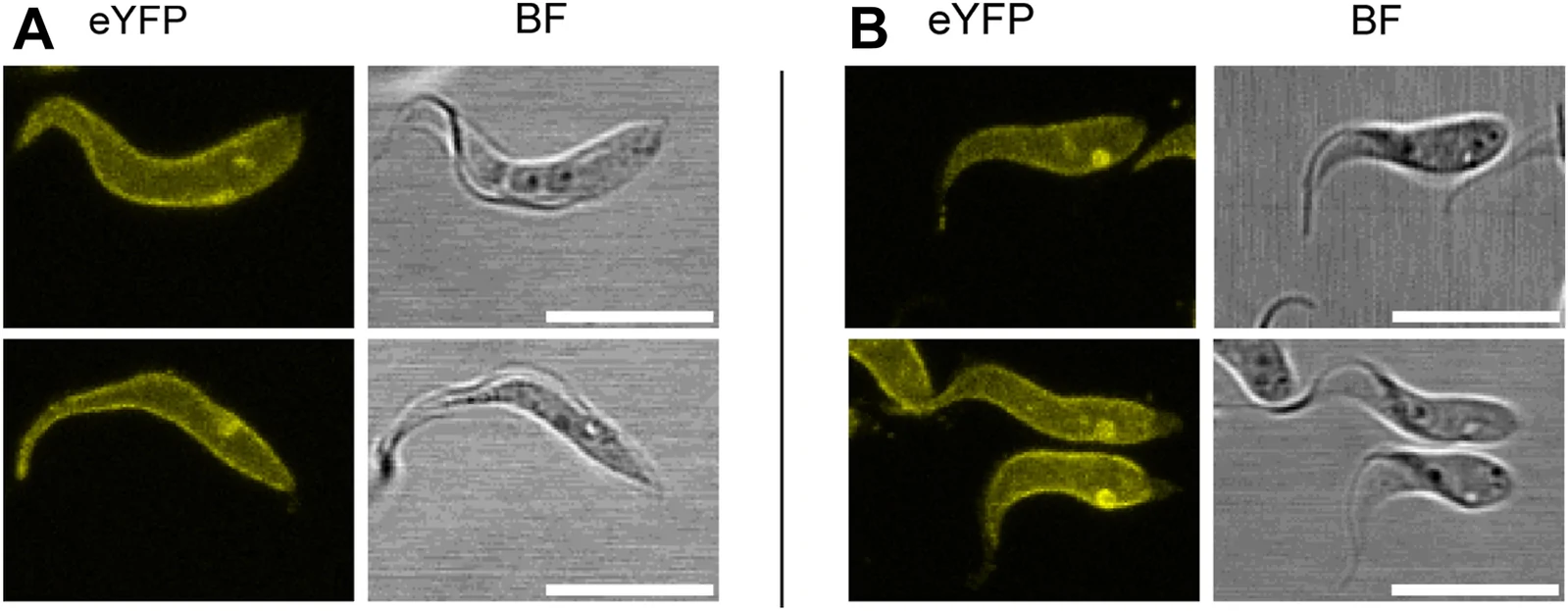
**AAT10.2 and Δ35-AAT10.2 localize at the plasma membrane of PCF *T*. *brucei*.** PCF *T. brucei* expressing N-terminally eYFP *in situ* tagged AAT10.2. *A*, native eYFP-AAT10.2 and (*B*) eYFP-Δ35-AAT10.2 visualized by confocal microscopy. eYFP fluorescence (*yellow*); scale bar 10 μm; BF, bright field.

The transport assays with *S. cerevisiae* cells showed BCAA uptake into the cell and thus supported the plasma membrane-localization of N-terminally *in situ* tagged mNeonGreen-AAT10.2 as reported in TrypTag for PCF *T. brucei* (56). The latter data not only showed localization of AAT10.2 at the plasma membrane, but also accumulation in the flagellar pocket. To verify these results under our growth conditions, N-terminal *in situ* eYFP-tagged AAT10.2 was localized in PCF *T. brucei*, which confirmed both the plasma membrane and flagellar pocket localization of AAT10.2 (Fig. 3*A*). Thus, the localization further supports a role of AAT10.2 in cellular uptake of BCAA.

As reported before on TrypTag for mNeonGreen (56) our attempts of tagging AAT10.2 with eYFP at the C-terminus failed. A tag may interfere with proper folding or trafficking when fused to the rather short (1–6 amino acids) predicted hydrophilic C-terminal region, but the reason of the failure was not further investigated.

### The AAT10.2 protein level is sensitive to BCAA concentrations in the medium

Haindrich *et al*. (30) reported upregulation of AAT10.2 mRNA levels in PCF *T. brucei* when starved of all amino acids for 2 or 6 h. Elevated mRNA levels under low amino acid availability may allow to adjust cellular levels of (essential) amino acids by modulating protein levels and transport activity according to metabolic needs. To determine whether AAT10.2 mRNA and protein levels are dependent on the BCAA concentration, in a first experiment PCF *T. brucei* lines with *in situ* eYFP-tagged AAT10.2 (eYFP-AAT10.2) were cultured in SDM79 (which contains approx. 300–400 μM of each BCAA), and in SDM79 supplemented with additional 2 mM of each BCAA. Immunoblot analyses showed a reduction of the AAT10.2 protein level at high BCAA concentrations in the medium (Fig. S8*A*). Furthermore, overexpression of 3xcMyc-tagged AAT10.2 showed that 3xcMyc-AAT10.2 mRNA levels did not show a decrease upon addition of 2 mM BCAA to the medium (Fig. S8*C*), while 3xMyc-AAT10.2 protein levels decreased (Fig. S2*B*), suggesting that AAT10.2 protein levels can be changed independent of mRNA level.

SILAC (stable isotope labeling with amino acids in cell culture) experiments were used to evaluate AAT10.2 phosphorylation at different BCAA levels. Because the AAT10.2 protein levels decrease at elevated BCAA concentrations in the medium, a decrease in the number of phosphopeptides is expected. When assessing the degree of phosphorylation relative to the non-phosphorylated protein, we found that AAT10.2 phosphorylation increased slightly from cells cultivated in 0.04 mM BCAA to cells cultivated in 0.4 mM BCAA. This increase was statistically significant for several phosphosites and one phosphopeptide (Table S4, Data S3, and S4). Further elevating BCAA to 5 mM resulted in only a marginal, non-significant increase. Because the measured differences are at the threshold of being recognized as changes, some uncertainty about the impact of the modifications remained. Several other proteins showed significant changes in phosphorylation under these conditions (log2-fold change >1, *p*-value <0.05), but no direct link to BCAA metabolism was evident (Datas S3 and S4).

As mentioned earlier, the changes in AAT10.2 protein levels seemed independent of mRNA abundance and primarily due to post-translational regulation. We further explored whether phosphorylation at the N-terminal domain of AAT10.2 might contribute to regulation in response to BCAA. To this end, mutated AAT10.2 variants were integrated into the native locus of AAT10.2 in the AAT10.2 RNAi cell line, thereby replacing the endogenous gene of one allele and preserving the native 3′UTR and its effect on mRNA stability. These different variants include the native protein (native), a version lacking the first 35 amino acids (Δ35), a phosphomimetic variant in which the highly phosphorylated serine residues 4, 6, 8, 11, 14, 19 and threonine 35 (*i*.*e*. all phosphorylation sites identified with a probability of >95% in our study; Table 1 and Fig. S9*A*) were replaced by aspartate (STD), and a non-phosphorylatable version in which the same serine and threonine residues were replaced by alanine (STA). To be able to assess the AAT10.2 protein level, the Ty-epitope was added at the N-terminus. All these (3xTy-tagged) AAT10.2 variants possess an altered codon usage - denominated as AAT10.2∗ - to allow expression in the RNAi lines, in which endogenous AAT10.2 can be downregulated by addition of tetracycline.

**Figure 4:**
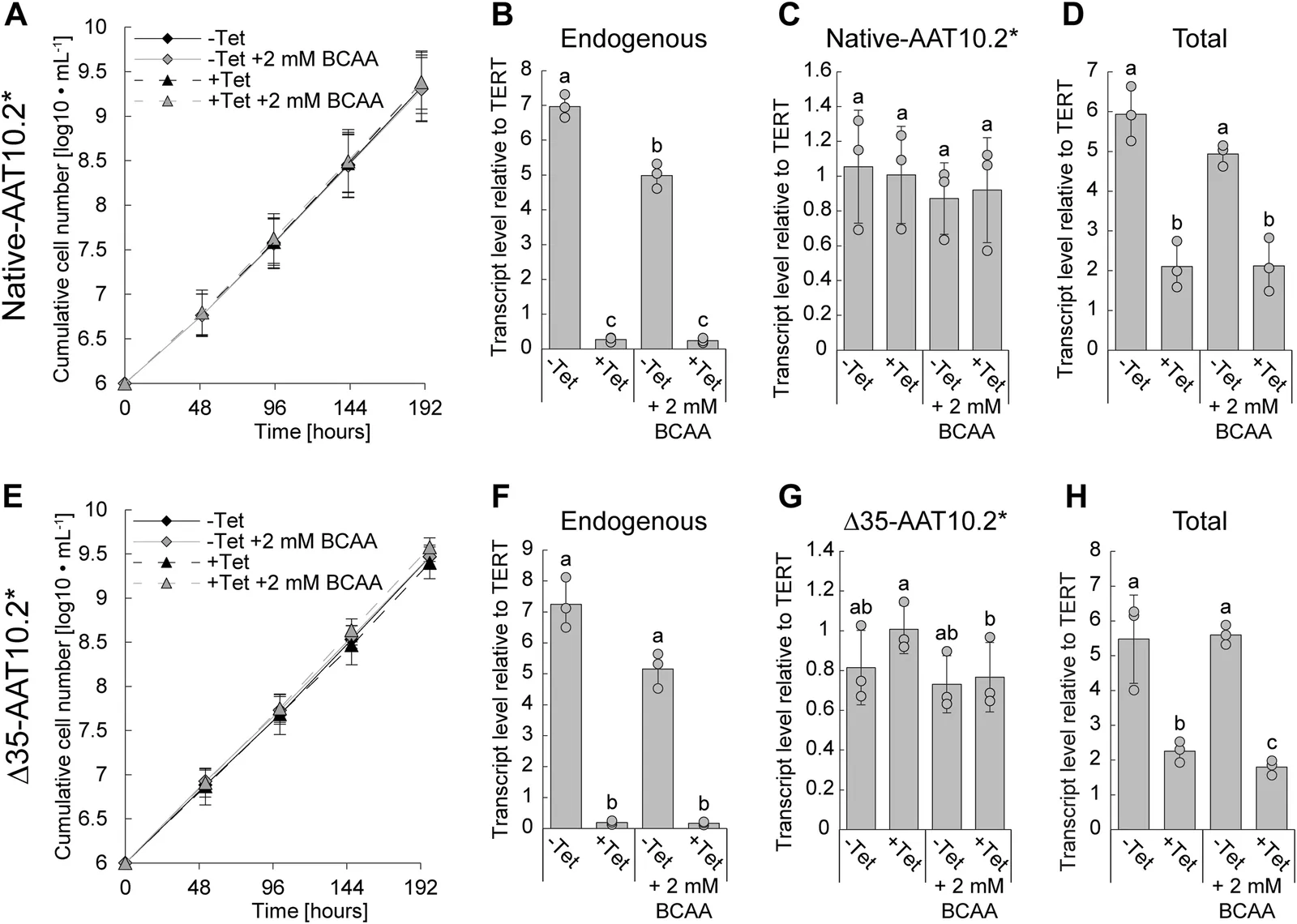
**Expression of tagged and mutated versions of 3xTy-AAT10.2∗ restores the growth defect of induced AAT10.2 RNAi cells.** Cumulative cell growth in SDM79 and associated mRNA levels of (*A–D*) 3xTy-AAT10.2∗ and (*E–H*) 3xTy-Δ35-AAT10.2∗ expressed in an AAT10.2-RNAi cell line in the absence of tetracycline (-Tet) or after induction (+Tet) of AAT10.2 RNAi and supplemented or not with 2 mM BCAA. mRNA levels determined after 72 h of RNAi induction of (*B* and *F*) endogenous AAT10.2, (*C*) 3xTy-AAT10.2∗, (*G*) 3xTy-Δ35-AAT10.2∗, and (*D* and *H*) total AAT10.2 mRNA (*i*.*e*. codon altered AAT10.2∗ plus endogenous AAT10.2). mRNA levels are given relative to TERT. Compact letters indicate statistically significant differences between treatments, error bar represent standard deviation (*p*-value<0.05, two-tailed *t* test, n = 3).

Variants 3xTy-AAT10.2∗ (native) and 3xTy-Δ35-AAT10.2∗ (Δ35) were able to complement the growth defect of the induced AAT10.2 RNAi line (3xTy-AAT10.2∗ Fig. 4*A*; 3xTy-Δ35-AAT10.2∗ Fig. 4*E*). RNAi efficiency and transcript levels of endogenous AAT10.2 and of 3xTy-AAT10.2∗ variants were evaluated by qRT-PCR 72 h after RNAi induction by utilizing three primer sets, one for monitoring the remaining endogenous allele (AAT10.2), one for the different Ty-tagged variants with altered codon usage (3xTy-AAT10.2∗), and a final set for monitoring total AAT10.2 mRNA (*i*.*e*. AAT10.2 plus 3xTy-AAT10.2∗) (Figs. 4, *B*–*D* and *F–H* and S9, *B* and *G*). Endogenous AAT10.2 transcripts were strongly downregulated upon mRNAi induction with tetracycline (Figs. 4, *B* and *F* and S9, *B* and *E*), while 3xTy-AAT10.2∗ transcripts levels of native, Δ35 and STA and STD variants remained largely unaltered (Figs. 4, *C* and *G* and S9, *C* and *F*). After induction of AAT10.2 RNAi, approximately half of the total AAT10.2 mRNA remained (Figs. 4, *D* and *H* and S9, *D* and *G*), indicating that indeed only the endogenous allele was downregulated. Addition of 2 mM BCAA slightly reduced endogenous AAT10.2 transcript levels (Fig. 4, *B* and *F*), and had only a small effect on 3xTy-AAT10.2∗ transcripts levels, that was in most cases not statistically significant (Fig. 4, *C* and *G*).

**Figure 5:**
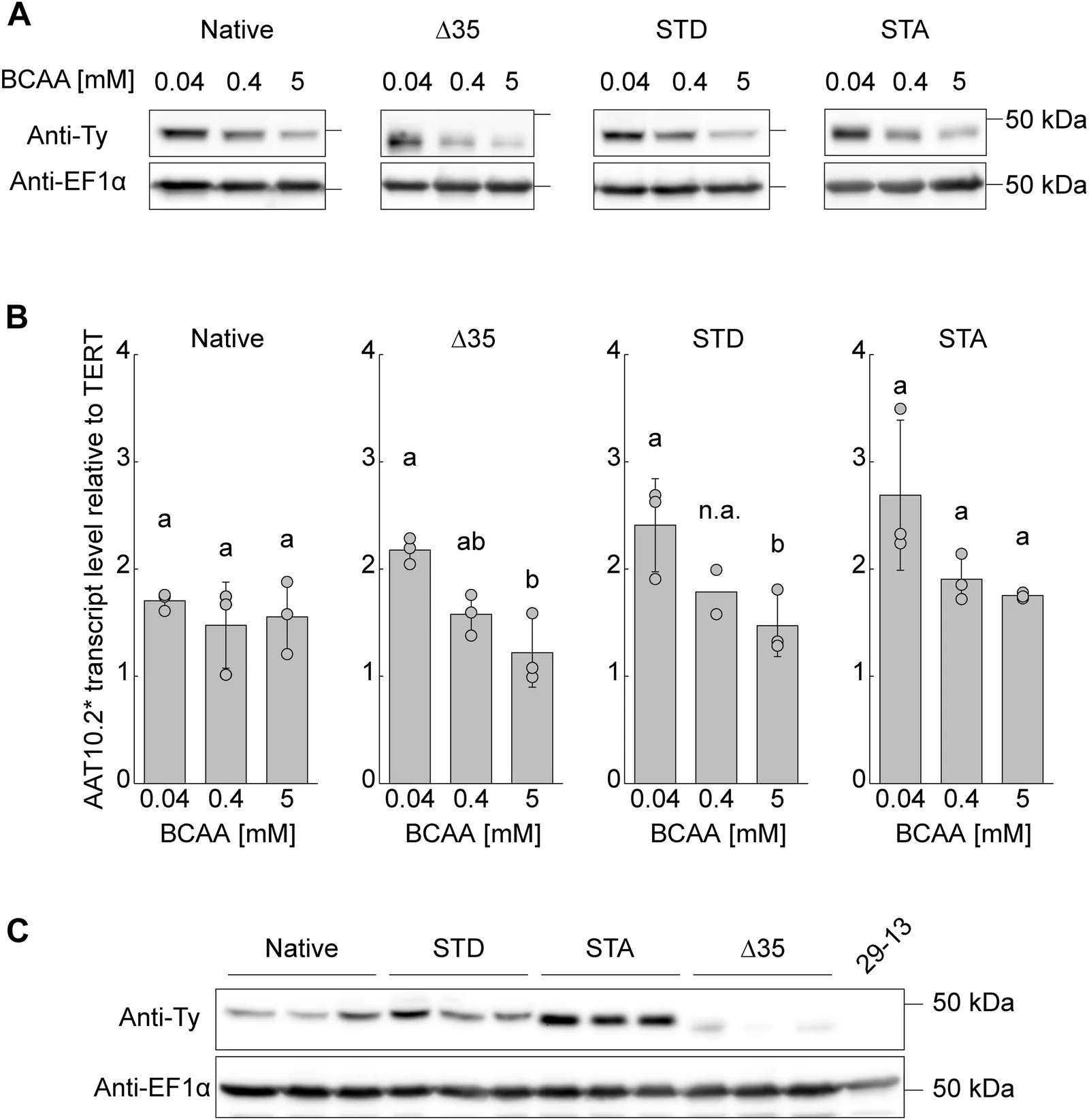
**AAT10.2 protein and mRNA levels decrease with increasing BCAA concentration in the medium and by removing the N-terminal hydrophilic region of AAT10.2.***A*, immunoblot analysis using induced AAT10.2 RNAi lines expressing 3xTy-AAT10.2∗ (native), 3xTy-Δ35-AAT10.2∗ (Δ35), 3xTy-STD-AAT10.2∗ (STD), or 3xTy-STA-AAT10.2∗ (STA) 24 h after changing the BCAA concentration in medium SDM79S from 0.4 mM to 0.04, 0.4, or 5 mM BCAA. EF-1α was used as control. *B*, mRNA levels of codon-altered AAT10.2∗ at 0.04, 0.4, and 5 mM BCAA relative to TERT. Compact letters indicate statistically significant differences between treatments; error bar represent standard deviation (*p*-value<0.05, two-tailed *t* test, n = 3); for STD at 0.4 mM BCAA n = 2, n.a. not analyzed. *C*, immunoblot analysis of 3xTy-AAT10.2∗ variants expressed in the induced AAT10.2 RNAi line; *i*.*e*. 3xTy-AAT10.2∗ (native), 3xTy-Δ35-AAT10.2∗ (Δ35), phosphomimicking mutant (STD), and non-phosphorylatable mutant (STA). Cells are from the same experiment as in *A* and *B*. For each variant 3 different clones (5 × 10^6^ cells) were used. Protein was detected using anti-Ty or anti-EF-1α antibodies.

To be able to better mimic starvation, in subsequent experiments the semi-defined medium SDM79S (30) was used, which was supplemented with different concentrations of BCAA. As shown for eYFP-AAT10.2 and overexpressed 3xcMyc-AAT10.2 before (Fig. S8), protein levels of all 3xTy-AAT10.2∗ variants showed a BCAA-dependent, gradual decrease, with lower 3xTy-AAT10.2∗ protein levels at the highest BCAA concentration (Figs. 5*A* and S10*A*). The pattern remained comparable regardless of mutations or truncation, suggesting that neither the N-terminal domain of AAT10.2 nor the phosphorylation sites play a direct role in regulation by BCAA. qRT-PCR showed that concomitantly, mRNA levels were generally highest at the lowest (0.04 mM) concentrations of supplemented BCAA, while at high BCAA levels, total AAT10.2 mRNA variants decreased, though this decrease was not statistically significant in all cases (Figs. 5, *A* and *B* and S10*B*).

Semi-quantitative comparison of AAT10.2 proteins levels in induced AAT10.2 RNAi lines expressing the different 3xTy-AAT10.2∗ variants and cultured in SDM79S with 0.4 mM BCAA (or in SDM79) showed strongly reduced 3xTy-Δ35-AAT10.2∗ protein levels and increased 3xTy-STA-AAT10.2∗ protein levels compared to 3xTy-AAT10.2∗ and 3xTy-STD-AAT10.2∗ (Figs. 5*C* and S10*C*). This pattern was conserved when cells were transferred to 0.04 mM or 5 mM BCAA (Fig. S11*B*). At 0.4 mM BCAA total AAT10.2 mRNA levels of the variants are in the same range (Figs. 5*B* and S11*A*) and thus can not explain the difference in 3xTy-AAT10.2∗ protein levels, suggesting that decreased protein stability or higher protein turnover of the truncated version and increased stability or reduced turnover of the non-phosphorylatable STA version may contribute to these differences.

**Figure 6:**
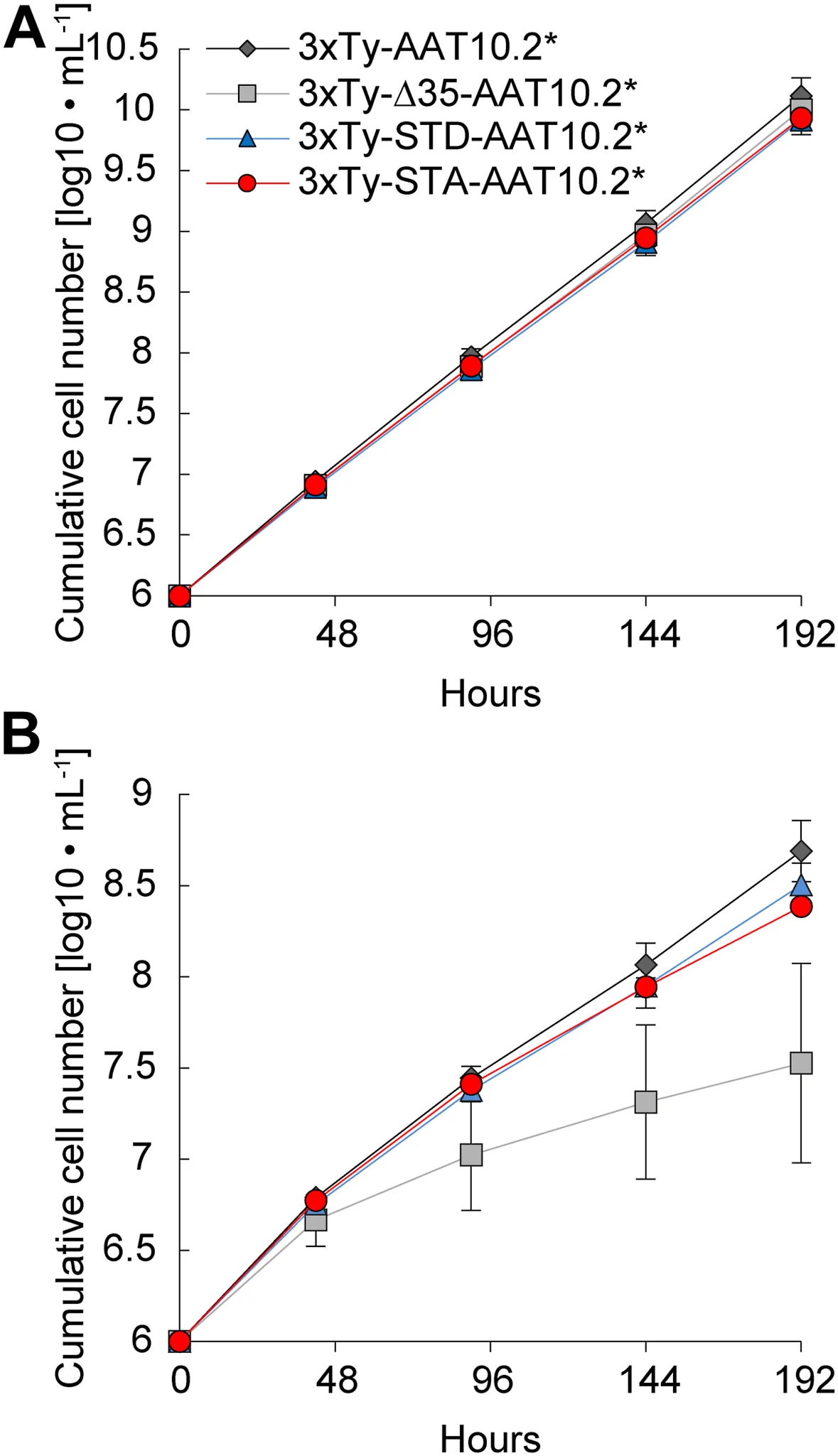
**Truncation of the N-terminal domain reduces cell growth at low BCAA availability.** Cumulative cell growth of induced AAT10.2 RNAi lines expressing 3xTy-AAT10.2∗, 3xTy-Δ35-AAT10.2∗, 3xTy-STA-AAT10.2∗, or 3xTy-STD-AAT10.2∗, cultured in SDM79S and supplemented either with (*A*) 0.4 mM or (*B*) 0 mM BCAA (n = 3, ±SD).

While protein levels of 3xTy-Δ35-AAT10.2∗ were reduced (Figs. 5*C* and S10*C*), no differences in growth were observed upon RNAi induction in SDM79S with 0.4 mM BCAA (Fig. 6*A*). Similarly, all variants performed comparably at very high (15 mM) BCAA, despite showing reduced growth (Fig. S12). In SDM79S without supplemented BCAA (labelled as “0 mM” BCAA, but still containing 7–17 μM BCAA from the 10% FBS (57)), induced *T. brucei* AAT10.2-RNAi cell lines expressing 3xTy-AAT10.2∗, 3xTy-STD-AAT10.2∗ or 3xTy-STA-AAT10.2∗ continued to grow slowly, but steadily, but cell growth of 3xTy-Δ35-AAT10.2∗ expressing lines was strongly reduced (Fig. 6*B*). In agreement with the hypothesis that altered protein levels is the main reason for decreased growth, other properties remained unchanged, *e*.*g*. similar to the non-truncated protein, eYFP-Δ35-AAT10.2 localized at the plasma membrane and flagellar pocket (Fig. 3*B*) and affinity and selectivity of Δ35-AAT10.2 expressed in the *S. cerevisiae* mutant YDR544 remained comparable to properties determined for AAT10.2 (Fig. S4). This suggests that the N-terminal domain of AAT10.2 does not play a role in localization and transport activity *per se*. To understand how the AAT10.2 RNAi cell lines expressing 3xTy-Δ35-AAT10.2∗ or 3xTy-AAT10.2∗ manage their amino acid pool during starvation for BCAA, the cellular content of amino acids was analyzed in parasites cultivated for 24 h and 72 h in SDM79S in the absence (“0 mM”) and presence (0.4 mM) of supplemented BCAA (Table S5, *A* and *B*). Similar to changes in amino acid levels after induction of RNAi (Table S3), levels of valine, leucine and isoleucine were lower when reducing supplemented BCAA to 0 mM for 24 h or 72 h, though for the truncated version the reduction was not in all cases statistically significant. In agreement with a difference in 3xTy-AAT10.2∗ protein levels between native and truncated versions (Fig. 5*C*), when supplemented with 0.4 mM BCAA, the amount of BCAA was generally lower in 3xTy-Δ35-AAT10.2∗ expressing cell lines than in cells expressing native 3xTy-AAT10.2∗, but differences were not always statistically significant (Table S5). When no BCAA was supplemented, the BCAA pool was further reduced and became comparable between the native and truncated versions, and BCAA levels remained in the same range during the experiment (24 h, 72 h). This suggests that the BCAA pool cannot be further reduced and may be maintained at the expense of a reduced growth rate (Table S5 and Fig. 6).

Taken together, the results suggest that the N-terminal domain does not determine affinity or selectivity of AAT10.2, but truncation affects the steady-state protein levels of AAT10.2 and, as a consequence, the BCAA content. At “0 mM” BCAA, the internal BCAA pools became comparable, but growth of cells expressing the truncated version showed a stronger defect, suggesting that when BCAA are scarce higher transport rates become essential.

## Discussion

### AAT10.2 is the main BCAA transporter in PCF *T. brucei*

Transport of BCAA in *T. brucei* has previously not been reported, while transport studies using intact *T. cruzi* cells indicated that a single transport system is responsible for the uptake of all three BCAA (58), though the gene encoding the *T. cruzi* BCAA transporter has not been identified yet. Comparable to the physiological data from *T. cruzi*, our results on AAT10.2 function in *T. brucei* and when expressed in *S. cerevisiae* support high selectivity also of the *T. brucei* BCAA transporter. On the one hand downregulation of AAT10.2 in PCF parasites exclusively decreased levels of leucine, isoleucine and valine and on the other hand, AAT10.2 expressed in *S. cerevisiae* not only supported direct uptake of leucine, isoleucine, and valine, but these amino acids also competed most strongly with the transport of leucine and isoleucine (Figs. 2*C*, S3, and S4), thus underlining narrow substrate selectivity of BCAA transport by AAT10.2 in *T. brucei*. While L-leucine transport in *T. cruzi* was inhibited by D-leucine (58), the inability of D-leucine to inhibit the uptake of L-isoleucine in *S. cerevisiae* cells expressing AAT10.2 suggests that transport in *T. brucei* is stereoselective. Furthermore, norleucine, a branched-chain amino acid with a 4-carbon side chain but lacking the methyl branch, showed low competition for isoleucine transport, highlighting the importance of a branched side chain. While substrate selectivity of AAT10.2 and BCAA transport in *T. cruzi* cells are–with the exception of D-leucine–very similar, the gene(s) encoding BCAA transporter(s) in *T. cruzi* cannot be identified based on phylogenetic relationship to AAT10.2, because AAT10.2 belongs to a *T. brucei* specific subclade shared with ornithine (28), proline/alanine (30) and several non-characterized transporters, and no very closely related gene can be found in the genomes of *T. cruzi* or *Leishmania* (18).

When expressed in *S. cerevisiae*, AAT10.2 was shown to be active over a pH range from 5.5 to 10.5. According to Liniger *et al*. (4), the pH in different compartments of the tsetse fly digestive tract varies between pH 7.9 and 10.6 and also depends on the time after a blood meal. The pH in the salivary glands was also reported to be slightly alkaline, *i*.*e*. pH 8 and pH 7.6 in freshly emerged and 15-day-old tsetse flies, respectively (59). AAT10.2 transcript levels are highest in midgut-derived parasites, *i*.*e*. 15 days post infection (5). According to Rose *et al*. (60), 15 days after infection corresponds to the establishment of *T. brucei* in the ectoperitrophic space and the differentiation into epimastigotes in the proventriculus, *i*.*e*. the stage before the parasites migrate to the salivary glands. Although so far the pH cannot be determined very precisely in these compartments, the broad pH range over which AAT10.2 is active (Fig. 2*B*) enables BCAA transport *via* AAT10.2 in probably all compartments, tissues and organs invaded during the life cycle of *T. brucei*.

The only moderate decrease in transport activity at increasing pH called into question the transport mechanisms and the role of protons as co-substrates. As mentioned before, members of the AAAP family were reported to move substrates in co-transport with protons or sodium (21, 23, 52). Leucine accumulation against the concentration gradient (when expressed in yeast) as well as BCAA-evoked inward currents measured in oocytes expressing AAT10.2 argued in favour of a symport rather than a uniport mechanism, most likely a co-transport of cations. Furthermore, the inability of sodium and potassium to increase transport rates when tested in *S. cerevisiae*, and the dependence of leucine transport on protons when expressed in oocytes, suggest that protons are the most likely co-substrate (Figs. S5 and S6). However, direct proof is missing because the low currents did not allow the measurement of proton flow with a proton-sensitive electrode. It therefore remains unclear whether, at high external pH, transport can be uncoupled from the co-substrate, whether the BCAA concentration gradient drives import into the cell, or whether local acidification is sufficient to sustain transport. For PCF *T. brucei* cultivated on plates, local acidification by half a pH unit was demonstrated, supporting the ability to increase extracellular proton availability (61). The presence of *T. brucei* in high-density swarms in the tsetse fly (62) may facilitate local acidification and the formation of a pH gradient. This local pH may differ from the pH in bulk solution and in non-infected insect tissues.

Though AAT10.2 may work in the reverse mode in the absence of a pH gradient (Fig. S7), there is so far no evidence for reverse transport in *T. brucei*, because BCAA concentrations were higher inside the cells than in the medium (Table S3), parasite growth at high BCAA is reduced (Fig. S12) and AAT10.2 protein abundance is decreased at high substrate levels.

Growth reduction after depletion of AAT10.2 by RNAi associated with a decrease in BCAA levels and rescue of growth when BCAA was supplemented showed that AAT10.2 is essential and the main BCAA transporter in PCF *T. brucei*. However, the fact that excess BCAA was able to rescue growth of induced AAT10.2 RNAi lines also suggests that there is either at least one additional BCAA transporter or that residual AAT10.2 transcript levels were sufficient to support growth. When assuming that biochemical properties in parasites and in the heterologous system are largely conserved and that in the parasite AAT10.2 does not display a dual affinity, compensation by one or several other transporter(s) seems more likely, though it is currently unknown which transporter supports BCAA uptake under these conditions. From the AATs characterized so far, only AAT6 may contribute to BCAA uptake, as it has been shown to transport a range of neutral amino acids with low apparent affinity when expressed in oocytes (27). AAT6 is higher expressed in BSF than PCF *T. brucei* (5), but eflornithine resistance assays showed that AAT6 is functional in PCF parasites (27). But while valine and isoleucine induced high currents, leucine was not a good substrate of AAT6. Furthermore, a methionine transport system characterized by measuring uptake of methionine into intact *T. brucei* parasites (63, 64) may support low affinity BCAA transport, though the respective gene has not been identified yet. This high affinity methionine uptake system (*K*_m_ approx. 30 μM) was shown to be rather selective for methionine and homocysteine, however the aromatic amino acids phenylalanine and tryptophan as well as leucine were also recognized, though they showed weaker competition (64). Such (low affinity) transport systems may replenish BCAA at high concentrations in the medium without sustaining the needs of *T. brucei* at low BCAA availability. However, so far only few AATs have been functionally characterized and (low affinity) transport of BCAA may also be supported by one or several other AAT members.

### AAT10.2 affects BCAA levels and *vice versa*

While cellular amino acid levels are somewhat variable between experiments, interestingly the changes in amino acid levels between starved PCF cells (“0 mM” BCAA) and cells cultured in 0.4 mM BCAA (for both 3xTy-Δ35-AAT10.2∗ and 3xTy-AAT10.2∗, Table S5) were largely comparable to the differences in amino acid concentrations between induced and non-induced AAT10.2 RNAi lines (Table S3) as shown by the ratio of individual amino acids. While BCAA were reduced, the aromatic amino acids, and in some cases methionine, lysine, and arginine levels increased. This implies that depletion of AAT10.2 and BCAA starvation result in a comparable shift of the amino acid pools (Tables S3 and S5).

These changes in amino acids may originate from increased demands of precursors by certain pathways that become essential under starvation, which may lead to increased or decreased levels of precursors. For example, methionine, apart from being essential for protein synthesis and as a methyl group donor for transmethylation of lipids and proteins (65), is also required for polyamine synthesis (66). Polyamines are used, for example, for the synthesis of trypanothione (67, 68), a key reducing agent that is essential for defense against oxidative stress (69). Trypanothione levels were not analyzed in our experiments, but an increased flow of methionine towards trypanothione may help in starvation-induced stress defense. Furthermore, the elevated levels of aromatic amino acids may feed into this pathway, as they were shown to participate in methionine recycling by acting as amino group donors (8, 9). Evidently, when analyzing and comparing amino acid levels under BCAA starvation or in AAT10.2 RNAi lines, we can currently only speculate on potential reasons for the changes observed, because modifications in amino acid pools might be due to shifts in utilization by biosynthetic pathways that build on amino acids as precursors, in differential degradation or biosynthesis, compartmentation or in changes of import or export rates. Furthermore, the changes in amino acid pools may not necessarily be due to altered flux in distinct metabolic pathways, but may alternatively result from upregulation of transporters – other than AAT10.2 – that recognize BCAA, but at the same time import other amino acids. A potential role of AAT6 (27) or a methionine uptake system (64) in low affinity BCAA transport have been discussed above. AAT6 would also support import of methionine and the aromatic amino acids phenylalanine and tryptophan (27). Also, the high affinity methionine transport activity characterized in *T. brucei* showed competition not only by leucine, but also by the aromatic amino acids phenylalanine and tryptophan (64). Higher abundance of such a methionine transporter may allow increased low affinity uptake of leucine, but may concomitantly result in elevated levels of methionine and aromatic amino acids. Therefore, upregulation of such (low affinity) transporters may partially explain the altered amino acid pools. However, at least on mRNA level, no upregulation of AAT6 was observed upon starvation of amino acids (30), while the gene encoding the methionine transporter is unknown and regulation is thus unclear. Interestingly, the concept of transporting non-polar amino acids like branched-chain and aromatic amino acids seems common and was shown for example, for human LAT4, which recognises phenylalanine and methionine in addition to BCAA (37, 70) and also in *S. cerevisiae* low- and high-affinity transporters of branched-chain and aromatic amino acids were described (71, 72).

At last, in the hemolymph of the tsetse fly BCAA concentrations are relatively high, though values vary between different reports (0.66–3.8 mM Leu, 0.9–9.4 mM Val, 0.67–3.8 mM Ile (3, 73, 74), while concentrations in SDM79 are much lower (300–400 μM). Information on amino acid pools in other compartments of the tsetse fly in which *T. brucei* proliferate is scarce. Bursell (73) demonstrated that three days after a blood meal the level of BCAA as well as phenylalanine and tyrosine decreased drastically in the thorax, and most likely also in other organs. Given the ability of PCF *T. brucei* to regulate the AAT10.2 protein level and to maintain the BCAA pool at the cost of growth, it may be suggested that *T. brucei* experiences short-term BCAA starvation during its development in the tsetse fly.

In contrast to PCF parasites, AAT10.2 was previously reported not to be essential for *in vitro* cultured BSF *T. brucei* and downregulation did not result in decreased BCAA levels (28). Reduction of AAT10.2 mRNA levels in induced AAT10.2 RNAi lines was only slightly less efficient in BSF (90%) (28) than in PCF (95%) *T. brucei*. The HMI-11 medium (75) used for BSF *T. brucei* cultures contains a twofold higher concentration of valine than the SDM79 medium (76) used for PCF parasites, while leucine and isoleucine concentrations are similar, indicating no major difference in BCAA availability. Furthermore, AAT10.2 transcript levels are lower in BSF parasites than in parasites extracted from flies (5) or *in vitro* cultured PCF *T. brucei* (77). Thus, it remains unclear whether BCAA requirements differ between BSF and PCF *T. brucei*, whether stronger reduction of AAT10.2 might also result in a phenotype in BSF *T. brucei* or whether additional transporters are involved in BCAA import in *in vitro* cultured BSF parasites.

### AATs are highly phosphorylated in the hydrophilic N-terminal domain

All AATs of the AAAP family identified in the phosphopeptide-enriched fractions were phosphorylated exclusively in their predicted hydrophilic N-terminal domain (Table 1). Similarly, phosphoproteome analyses of *T. cruzi* and *Leishmania infantum* (78, 79, 80), reported phosphorylation of AATs of the AAAP family exclusively in their hydrophilic N-terminal domains, and identified phosphorylated N-terminal peptides of the lysine transporter AAP7 (Tc00.1047053511545.80) and the proline/alanine transporter AAP24 (Tc00.1047053504069.120) in *T. cruzi*, as well as the two AAT20 transporters (LinJ.10.0760 and LinJ.10.0770), two AAT1 proteins (also named AATP11; LinJ.31.0350/LinJ.31.0360) and one putative amino acid permease (LinJ.27.2680) in *Leishmania*. This suggests that phosphorylation of the hydrophilic N-terminal region might be an intrinsic feature shared among AAAPs of trypanosomes.

Common among all trypanosomatid AATs is that their N-terminal hydrophilic domain is highly variable (81) and predicted to have a disordered structure (49), thus enabling high variability in conformations. In *Leishmania*, 18 amino acids of the N-terminal domain of the proline and alanine transporter LdAAP24 are essential for alanine transport activity of AAP24, but not for proline transport (82). It appears that this domain is highly negatively charged. The authors speculated on the possible role of this negatively charged domain, which may, for example, determine the tertiary structure and/or interact with the slightly positively charged loop regions. While also in AAT10.2 internal loops are slightly positively charged, the negative charges of the N-terminus are very different; in *Leishmania* AAP24 the mean charge of the N-terminal hydrophilic tail at pH 7 is −11.8, while in *T. brucei* AAT10.2 it is −1.9. However, in AAT10.2, phosphorylation may provide additional negative charge. Evidently, not all *Leishmania* AATs have a very strongly negatively charged N-terminal hydrophilic domain, and for *T. brucei* AATs the number of distinct phosphorylation sites varies. Furthermore, the role of the extra negative charge may differ in different AATs.

When increasing the BCAA concentration, AAT10.2 protein levels decrease; concomitantly, in SILAC experiments the number of phosphorylated AAT10.2 peptides also decreased. Relative to non-phosphorylated AAT10.2, there is a tendency toward an increase in AAT10.2 phosphosites and in one phosphopeptide at elevated BCAA levels, but there is no massive or prominent change. This suggests that either (*i*) more phosphorylation leads to greater degradation, thereby preventing the further accumulation of phosphorylated AAT10.2, or high overall phosphorylation levels of AAT10.2 mask additional phosphorylation, or (*ii*) phosphorylation does not play a role in AAT10.2 protein abundance. Although elevated levels of the STA-AAT10.2∗ variant suggest a role of phosphorylation in regulating protein levels, the fact that protein abundance of all variants decreased upon addition of BCAA does not support a role of phosphorylation in substrate-induced degradation.

Multiple examples of charge- or phosphorylation-based regulation of transporters have been documented (37, 39, 41, 83, 84, 85, 86, 87, 88). However, at least when expressed in yeast, selectivity and apparent affinity of AAT10.2 did not change when removing or mutating the N-terminal domain (Figs. S4 and S13), arguing that transport activity is not directly affected by phosphorylation. In *T. brucei*, the BCAA pool was decreased in AAT10.2 RNAi lines expressing N-terminally truncated Δ35-AAT10.2∗ at 0.4 mM BCAA (Table S5), but this reduction may most easily be explained by the decrease in the Δ35-AAT10.2∗ protein level (Fig. 5*C*), rather than by altered transport rates. Similarly, when BCAA were limited, only cells expressing the truncated AAT10.2 showed a growth defect, while growth of phosphomimicking mutants was comparable to the native version (Fig. 6*B*).

The higher protein level of the STA-AAT10.2∗ variant compared to the native and the STD-AAT10.2∗ mutant (Fig. 5) suggested a role of the negative charge or phosphorylation in transporter destabilization. However, this role could not be experimentally confirmed. Despite higher protein levels in *T. brucei*, but comparable activity and apparent affinity when assessed *in S. cerevisiae* (Fig. S13), 3xTy-STA-AAT10.2∗ did not allow faster growth rates under limiting BCAA conditions (Fig. 6*B*) and was not more strongly reduced in growth at high BCAA (Fig. S12).

While phosphorylation seems to be a common modification, whether and how phosphorylation of the N-terminal hydrophilic region contributes to optimize amino acid acquisition by AAT10.2 or other AATs needs to be further explored. Evidently, distinct regulation may allow a more sophisticated nutrient management for different amino acids.

In summary, AAT10.2 was found to be an essential, plasma membrane-localized transporter for BCAA in PCF *T. brucei*. Transport is highly selective for BCAA, which are transported with high affinity. While the N-terminal region of AAT10.2 was found to be phosphorylated at multiple residues, the role of this modification still needs to be further elucidated. The phosphorylation of the hydrophilic N-terminal domain in other AATs also suggests that it plays a versatile role in regulation and interaction, which may ultimately facilitate adaptation.

## Experimental procedures

### Plasmid constructs

Plasmids for expression of AAT10.2 in yeast: Phosphomimetic mutants of AAT10.2, in which serine residues 4, 6, 8, 11, 14, and 19 are substituted by aspartate, were produced by amplifying the open reading frame of AAT10.2 from pDR197-AAT10.2 (28) by PCR using primers QO06 and 8300Bam (for sequence of all oligonucleotides, see Table S6). Similarly, using QOO7 and 8300Bam primers, the same serine residues were changed to alanine. After restriction with *Eco*RI and *Bam*HI the amplicons were inserted into pDR197 (89), thereby generating constructs pDR-SD-AAT10.2 and pDR-SA-AAT10.2, respectively. Threonine 35 was substituted by aspartate or alanine using the In-Fusion kit (Takara Bio USA, Inc). With primers QO30 and QO31, threonine 35 in pDR-AAT10.2 and pDR-SD-AAT10.2 was changed to aspartate, generating plasmids pDR-TD-AAT10.2 and pDR-STD-AAT10.2, respectively. With primers QO29 and QO31, threonine 35 was changed to alanine by using pDR-AAT10.2 and pDR-SA-AAT10.2 as templates, thereby pDR-TA-AAT10.2 and pDR-STA-AAT10.2 were made.

For truncated AAT10.2, *i*.*e*. Δ35-AAT10.2, the entire pDR-AAT10.2 plasmid (28) with exception of nucleotides 4 to 102 of the AAT10.2 open reading frame, was amplified by PCR using primers QO101 and QO102. *Dpn*I cleavage was used to remove the template plasmid. Subsequently, the product was purified using the NucleoSpin Gel and PCR Clean-up kit (Macherey-Nagel) and circularized with the pEASY-Uni Seamless Cloning and Assembly Kit (TransGen), following the manufacturer's instructions. This resulted in plasmid pDR-Δ35-AAT10.2.

For expression of AAT10.2 in *X. laevis* oocytes, AAT10.2 was excised from pDR197-AAT10.2 using the enzymes *EcoR*I and *Bam*HI and cloned into the *Eco*RI and *Bgl*II sites of the oocyte expression vector pBF1 (90), resulting in the plasmid pBF1-AAT10.2.

Plasmids for expression of AAT10.2 variants in *T. brucei*: To generate Ty-tagged AAT10.2, the first vector pPOTv6-Blast was generated by removing mNEO from pPOTv6-Ty-mNEO-Ty (91) by using primers QO62 and QO63, and reassembling using the pEASY-Uni Seamless Cloning and Assembly Kit (TransGen Biotech, Beijing, China). Subsequently, AAT10.2 was amplified by PCR with primers QO37 and 8300Bam from pDR197-AAT10.2 (28), purified by gel extraction, restricted with *Hind*III and *Bam*HI, and cloned into *Hind*III and *Bam*HI sites of pPOTv6-Blast using T4 DNA ligase (Biolab, New England), thereby generating pPOTv6-3xTy-AAT10.2. Similarly, the open reading frame (ORF) of STD-AAT10.2 was amplified using pDR-STD-AAT10.2 as template and primers QO57 and 8300Bam, and the STA-AAT10.2 ORF was amplified using pDR-STA-AAT10.2 and primers QO56 and 8300Bam. Both fragments were cloned into the *Hind*III and *Bam*HI sites of pPOTv6-Blast, resulting in pPOTv6-3xTy-STD-AAT10.2 and pPOTv6-3xTy-STA-AAT10.2, respectively.

Expression of AAT10.2 variants in the *T. brucei* AAT10.2 RNAi line: In order to express AAT10.2 and mutated versions in the AAT10.2 RNAi *T. brucei* line, nucleotides 153 to 564 of the AAT10.2 ORF were replaced by a fragment with altered codon usage. pUC-AAT10.2^790-CA^ was provided by GenScript and contains the first 790 bp of the AAT10.2 ORF, with altered nucleotides 153 to 564 (see Fig. S14). pUC-AAT10.2^790-CA^ served as template to generate different AAT10.2 versions (named AAT10.2∗) in which the codon usage in the region of the RNAi target sequence is altered. The region with altered codons was amplified by PCR from pUC-AAT10.2^790-CA^ with primers QO86 and QO87 for AAT10.2∗, STD-AAT10.2∗ and STA-AAT10.2∗ or primers QO81 and QO82 for Δ35-AAT10.2∗. The amplified fragments were purified by gel extraction. The vectors and a part of the inserts of pPOTv6-3xTy-AAT10.2, pPOTv6-3xTy-STD-AAT10.2, and pPOTv6-3xTy-STA-AAT10.2 were amplified by PCR, using primers QO84 and QO85. For the Δ35-AAT10.2∗ construct, vector pPOTv6-3xTy-AAT10.2 was amplified with primers QO80 and QO79. The template plasmid was cleaved with *Dpn*I and the amplicons cleaned by PCR cleanup (Nucloeospin, Macherey-Nagel). The amplified insert and vector fragments were ligated by using the pEASY-Uni Seamless Cloning and Assembly Kit (TransGen Biotech) resulting in plasmids pPOTv6-3xTy-AAT10.2∗, pPOTv6-3xTy-STD-AAT10.2∗, pPOTv6-3xTy-STA-AAT10.2∗ and pPOTv6-3xTy-Δ35-AAT10.2∗.

For inducible overexpression of 3xcMyc-tagged AAT10.2, the ORF was amplified with primers QO37 and 8300Bam from pDR197-AAT10.2 (28), purified by gel extraction, restricted with *Hind*III and *Bam*HI, and cloned into *Hind*III and *Bam*HI sites of pJM-2, which is based on pLew100 (92) and contains a puromycin resistance and a 3xcMyc cassette for N-terminal tagging (93), thereby generating pLew-3xcMyc-AAT10.2. All constructs were confirmed by sequencing.

### Isolation of genomic DNA

For isolating genomic DNA from *T. brucei* 1 × 10^7^ cells were pelleted at 1000*g* for 10 min and resuspended in 300 μL extraction buffer (0.2 M Tris-HCl pH 9.0, 0.4 M LiCl, 25 mM EDTA, 1% SDS), then 300 μL of phenol:chloroform:isoamyl alcohol (50:49:1) was added and mixed, and finally debris was pelleted by centrifugation for 5 min at 10,000*g*. The supernatant was transferred to a new tube containing 250 μL isopropanol, mixed and centrifuged for 30 min at 10,000*g* at 4 °C. The pellet was washed with 70% ethanol, dried, and DNA was solubilized in 50 μL distilled water.

To assess the replacement of the AAT10.2 locus by the different DNA constructs, genomic DNA was used as a template for PCR using primers QO68 and QO69. Integration was verified based on expected fragment size.

### *T.brucei* cell culture

PCF *T.* *brucei* 29-13 (92) or a single marker cell line (94) were cultured at 28 °C in standard medium (SDM79) (Bioconcept, Switzerland) (76), or in home-made medium SDM79S (30) supplemented with varying amounts of BCAA (as indicated in each figure). The media were supplemented with fetal bovine serum (FBS) (Gibco, Thermo Fisher Scientific) as specified below, 7.5 mg L^−1^ hemin and 15 mg L^−1^ folic acid.

PCF 29-13 cells were cultivated with 25 μg mL^−1^ hygromycin, 15 μg mL^−1^ neomycin and 10% FBS (v/v). Cell lines containing the AAT10.2 RNAi construct were additionally supplemented with 2.5 μg mL^−1^ phleomycin. Cells transfected with PCR fragments (from constructs in pPOTv4/v6) were selected and cultivated with additional 10 μg mL^−1^ blasticidin.

*T*. *brucei* single marker cells were cultured with 2.5 μg mL^−1^ phleomycin and 5% FBS (v/v), when containing 3xcMyc-AAT10.2, additionally 1 μg mL^−1^ puromycin was added. Expression of 3xcMyc-AAT10.2 and AAT10.2-RNAi was induced with 1 μg mL^−1^ tetracycline. Cell numbers in culture were measured with a Guava Muse Cell analyzer (Cytek Biosciences) and diluted to 1 × 10^6^ cell mL^−1^ every 48 h, to keep logarithmic growth (*i*.*e*. 1–10 × 10^6^ cells mL^−1^).

### Transfection of trypanosomes

Transfection of *T. brucei* was performed as described (95). Briefly, 2 × 10^7^ cells were harvested by centrifugation (10 min at 800*g*, room temperature) and washed once in modified cytomix as described by Niemirowicz *et al*. (95) at 4 °C. Subsequently, the cell pellet was resuspended in 450 μL modified cytomix and mixed with the DNA amplicons or linearized plasmids. The cell suspension was transferred into a 2 mm gap cuvette (Bio-Rad Laboratories) and electroporated (BTX600, 1.4 kV, 24 Ω). Pulsed cells were transferred to 10 mL SDM79 medium. Select antibiotics were added the next day; cells were diluted 1:5 and distributed (1 mL each) across 48-well plates.

The following plasmids or fragments were used to produce the different cell lines. AAT10.2 RNAi PCF (29-13) cell lines were generated by using plasmid pMS14-AAT10.2 (28). After digestion of the plasmid with *Not*I and purification *via* PCR clean-up, the linearized plasmid was used for transfection.

For gene-replacements or addition of tags, long-primer-PCR was performed (96), using pPOTv4 or the generated pPOTv6 constructs as templates. For replacement of AAT10.2 by 3xTy-tagged AAT10.2∗ variants, QO25 and QO89 primers were used for amplification of Blast-3xTy-AAT10.2∗, Blast-3xTy-STA-AAT10.2∗, Blast-3xTy-STD-AAT10.2∗ and Blast-3xTy-Δ35-AAT10.2∗ from the respective pPOTv6-constructs and the PCR product was used for transfection of the PCF *T. brucei* AAT10.2 RNAi line. For N-terminal *in situ* tagging of AAT10.2 with eYFP, pPOTv4-blast (91) served as template for PCR with primer QO25 and QO26. The PCR fragment was used for transfection to generate cell line eYFP-AAT10.2 (29-13). Similarly, QO25 and QO61 were used for generating *in situ* tagged eYFP-Δ35-AAT10.2.

For inducible overexpression of 3xcMyc-tagged AAT10.2, pLew-3xcMyc-AAT10.2 was linearized with *Not*I and used for transfection of single marker PCF *T. brucei* (87).

### *S. cerevisiae* transformation and transport assays

*S. cerevisiae* strain YDR544 (*Matα*, *ura3*-*1*, *gap1*-*1*, *put4*-*1*, *uga4*-*1*, *ssy1*::*kanMX*) (51) was transformed according to Dohmen *et al*. (97). Transformed yeast cells were selected on synthetic dextrose (SD) medium (1.7 g L^−1^ yeast nitrogen base without amino acids and without ammonium sulfate (Difco, BD Biosciences, Franklin Lakes, NJ, USA), supplemented with 20 g L^−1^ glucose, 5 g L^−1^ ammonium sulfate, and 20 g L^−1^ Bacto agar (Becton, Dickinson and Company, Sparks, MD USA). Transport assays were performed as described previously (98), with slight modifications. Briefly, transformed *S. cerevisiae* cells were cultivated in SD to an OD_600_ of approximately 0.6. The cells were then transferred to YPD medium (10 g L^−1^ bacto yeast extract, 20 g L^−1^ of bacto peptone, 20 g L^−1^glucose) for 1 h. The cells were harvested by centrifugation at 2500*g* for 5 min, washed with water, and diluted to an OD_600_ of 0.6. Subsequently, cells were pelleted and resuspended in 1/10 volume of buffer A (50 mM potassium phosphate at pH 7, 0.6 M sorbitol), and stored on ice. Prior to the transport assays, the cells were incubated for 5 min with 100 mM glucose at 30 °C. Alternatively, *i*.*e*. for determining pH dependence, cells were diluted to an OD of 0.8, pelleted and resuspended in 1/10 volume of 0.8 M sorbitol. Cells were placed on ice until use. Prior to the transport assays, the cells were diluted to OD 6 by adding 40 mM potassium phosphate and 40 mM boric acid, at pH as indicated (adjusted with KOH); then 100 mM glucose was added, and cells were incubated for 5 min at 30 °C. For ion dependencies, prior to the transport assays, cells at OD 8 were diluted to an OD 6 by addition of 50 mM potassium phosphate, 50 mM sodium phosphate or 50 mM TRIS-phosphate (adjusted to pH 7 with KOH, NaOH or TRIS, respectively), containing 0, 2, or 20 mM NaCl or KCl; then glucose was added and cells were incubated for 5 min at 30 °C. To start the transport assays, an equal volume of cells and substrate (in the same buffer as the cells) was mixed and incubated at 30 °C. To start the transport assays, an equal volume of cells and substrate (in the same buffer as the cells) was mixed and incubated at 30 °C. The substrate, contained varying concentrations of L-leucine, L-isoleucine or L-valine spiked with 56.9 to 113.8 kBq mL^−1^ L-[^3^H]-leucine (2.22 TBq mmol^−1^), 170.8 kBq mL^−1^ L-[^3^H]-isoleucine (1.85 TBq mmol^−1^) or 85.4 kBq mL^−1^ L-[^3^H]-valine (2.22 TBq mmol^−1^) (Hartmann Analytic, Braunschweig, Germany) per assay, and competing compounds as indicated in the figures. Amino acids refer to L-amino acids, only D-amino acids are specified. If not indicated otherwise, after 30, 60, 90, 120, and 150 s aliquots were transferred to 4 mL of ice-cold buffer A or modified buffer A (*i*.*e*. for determining pH dependence; 0.6 M sorbitol, 40 mM potassium phosphate, 40 mM boric acid, and pH as indicated), filtered on glass microfiber filters (Whatman grade GF/C), and washed twice with 4 mL of ice-cold buffer A (or modified buffer A). The filters were immersed in 4 mL of Ultima Gold XR (Revvity Health Sciences B.V., Groningen, Netherlands) scintillation cocktail, and samples were analyzed by liquid scintillation counting on a Tri-Carb 2910TR (PerkinElmer GmbH). Results are displayed as uptake rates calculated from the time series performed in each transport assay. Transport rates measured in cells transformed with the yeast expression vector, pDR197 (89), were subtracted. Calculation of kinetic parameters was done using nonlinear least squares regression and the Michaelis-Menten kinetic model. With exception of valine transport (Fig. S3) and kinetics of variants (Fig. S13), the results include at least three independent transformants, with at least one dataset collected in an independent experiment.

Concentrations of [^3^H]-leucine in *S. cerevisiae* cells were calculated for the 2.5 min time-points and based on a cell diameter determined under our growth condition (4.0–4.2 μm) and a simple sphere which, although slightly lower (37 μm^3^), corresponds to average cell volumes of approximately 40 to 50 μm^3^ found in other studies (99, 100). Transport rates were taken from Figure 2.

### Electrophysiology and efflux measurements in *X. laevis* oocytes

pBF1-AAT10.2 was linearized with *Mlu*I, and cRNA was synthesized using the SP6 mMessage mMachine transcription kit (Invitrogen/Thermo Fisher). For electrophysiology, *X. laevis* oocytes were obtained *via* partial ovariectomy (permission registered at the government of Lower Franconia, ref. No. 55.2.2-2532-2-1850-11). Oocytes were treated with collagenase I and washed using ND96 buffer solution (96 mM NaCl, 2 mM KCl, 1 mM MgCl_2_, 1 mM CaCl_2_, 10 mM HEPES, pH 7.4). Oocytes of stage V were injected with 50 nl (50 ng) cRNA, or 50 nl nucleotide free water using a Nanoject III microinjector (Drummond Scientific) and stored in ND96 solution supplemented with gentamycin (50 μg mL^−1^), at 16 °C for 4 to 5 days until measurements were performed. For efflux experiments, *X. laevis* oocytes of grade 1 were purchased from EcoCyte Bioscience, and injected with 50 nl (50 ng) cRNA, or 50 nl nucleotide-free water. Injected oocytes were stored in Barth’s solution (88 mM NaCl, 1 mM KCl, 0.33 mM Ca(NO_3_)_2_, 0.41 mM CaCl_2_, 0.82 mM MgSO_4_, 2.4 mM NaHCO_3_, 10 mM HEPES, pH 7.4) with gentamycin (50 μg mL^−1^) at 15.5 °C for 3 to 5 days until measurements were performed.

TEVC setup was essentially as described previously (101) and measurements were performed in modified Ringer solution (115 mM NaCl, 2 mM KCl, 1.8 mM CaCl_2_,1 mM MgCl_2_, 5 mM HEPES for pH 7 and 8 or 10 mM MES for pH 4, 5, 5.5 and 6). Pipettes were pulled to a resistance of 0.2 to 1.5 MΩ, and oocytes were clamped at a holding potential of −40 mV for single-pulse measurements. Voltage jumps were performed from an initial holding potential of 0 mV to 50 ms pulses between −140 and 0 mV in 20 mV increments. Steady-state currents (I_SS_) in the absence of substrate were measured before and after applying the respective substrate. Hence, substrate-induced I_SS_ were determined by subtracting currents in the absence of substrate from the currents in the presence of substrate. To determine the *K*_*M*_ value for protons, leucine-induced currents of individual AAT10.2-expressing oocytes were measured at 2 mM leucine and different pH values. The respective I_SS_ at −80 mV were calculated, normalized to *I*_max_ and plotted as a function of the proton concentration. The corresponding leucine-induced currents were fitted with the Michaelis Menten equation. All values represent the mean ± SD obtained from at least three different oocytes using at least two independent batches of oocytes.

For export experiments, 21 oocytes expressing AAT10.2 and water-injected oocytes were injected on ice with 10 pmol leucine containing 0.37 kBq L-[^3^H]-leucine (Hartmann Analytic). After 10 min recovery in ice-cold Barth’s buffer, oocytes were washed and batches of 3 oocytes were transferred to 2 mL Barth’s solution, pH 7.4 at room temperature (22–25 °C). At indicated time points batches of oocytes were transferred to ice-cold Barth’s buffer to stop the export followed by a second wash with ice-cold Barth’s solution. Individual oocytes were lysed in 100 μL 10% SDS for 15 min, mixed with 3 mL Ultima Gold XR (Revvity Health Sciences B.V., Groningen, Netherlands), and samples were analysed by liquid scintillation counting. For time point 0, oocytes were directly washed and lysed after the recovery period. Aliquots of the export buffers of each oocyte batch (and therefore timepoint) was also subjected to liquid scintillation counting. Experiments were repeated four times using oocytes from two different frogs.

### Immunoblotting

Cells were harvested by centrifugation at 1100*g* for 10 min at 4 °C and washed 2x in PBS (137 mM NaCl, 2.7 mM KCl, 10 mM Na_2_HPO_4_, 1.8 mM KH_2_PO_4_, pH 7.4) containing protease inhibitors (protease inhibitor cocktail tablets Roche, Basel, Switzerland) according to the manufacturer’s instruction, and 1 mM phenylmethylsulfonyl fluoride (PMSF, Sigma, Merck). Cell pellets were dissolved in Laemmli buffer (102) and incubated at 50 °C for 10 min and further mixed until complete solubilization. Proteins (corresponding to 5 × 10^6^ cells) were then separated by SDS-PAGE and transferred to PVDF membranes. Membranes were blocked with 5% non-fat milk in TBS (50 mM TRIS, 200 mM NaCl, pH 7.5), stained with 0.1% Ponceau S (Sigma, Merck, Sigma) in 5% acetic acid (in water), destained using TBS and subsequently incubated with primary antibody (1:4000, anti-Ty BB2, MA5-23513, Invitrogen; 1:2000, anti-GFP IgG1, 11814460001, Sigma, Merck; 1:1000 anti-cMyc 9E10, sc-40, Santa Cruz Biotechnology, Santa Cruz, CA, USA; 1:2000 anti-EF-1α, sc-21758, Santa Cruz Biotechnology) overnight at 4 °C. Elongation factor 1-alpha (EF-1α) was used as a loading control for Western blots. Membranes were washed three times with TBS-T (*i*.*e*. TBS containing 0.05% Tween 20) and incubated with a secondary antibody (horseradish peroxidase-conjugated goat anti-mouse IgG (Sigma)). Detection was performed using SuperSignal West Pico PLUS Chemiluminescent Substrate (Thermo Fisher) according to the manufacturer’s instructions. Signals were recorded using a ChemiDoc MP Imaging system (Bio-Rad Laboratories, Hercules). Representative images are shown. Western blot results displayed in the main text were verified in independent experiments, and these results are shown in supplementary figures. Images of entire blots can be found in Figs. S15–S19.

### Quantitative RT-PCR

RNA was isolated from 1 × 10^7^ cultured cells with TRI reagent (Merck KGaA, Darmstadt, Germany) according to the manufacturer’s instructions. RNA samples were treated with DNase I (Roche, Basel, Switzerland) for 1 h at 37 °C, followed by a phenol-chloroform extraction and ethanol precipitation. 0.5 μg RNA was used for cDNA synthesis (in 20 μl) with PrimeScript reverse transcriptase (TAKARA, Takara Bio), using random 6-mers and oligodT primers. Real-time quantitative PCR was performed with a Light cycler 96 (Roche). The reaction mixture consisted of TB Green premix (Takara Bio) and three primer sets: one to monitor the remaining wild type allele (QO95 and QO96), one to monitor the Ty-tagged and codon-altered allele (QO90 and QO91) and one for both, *i*.*e*. wild type and codon-altered alleles (8300qPCR_F and 8300qPCR_R). The q(RT)-PCR were performed with 1 or 2 different cDNA dilutions (1:100 and 1:200), and in technical duplicates; average values of the two or four values were used as one data point. Telomerase reverse transcriptase (TERT, Tb927.11.10190 (103), or AN1-like zinc finger (AN1, Tb927.10.12970, (104, 105)) were used as reference genes. The absence of DNA contaminants was verified by using samples lacking reverse transcription. Each qRT-PCR was repeated with RNA from 3 different clones (*i*.*e*. biological replicates) for each cell line, unless mentioned otherwise. Statistical significance was determined by using paired two-tailed t-tests for comparison of different growth conditions and unpaired two-tailed t-tests for comparison between different variants.

### Fluorescence live cell imaging

For localization of eYFP in *in situ* tagged AAT10.2 and Δ35-AAT10 cell lines, cells were harvested by centrifugation at 800*g* for 10 min and washed with PBS. Subsequently, cells were resuspended and washed with PBS containing 50 mM sucrose and concentrated to a density of 1 × 10^8^ cells mL^−1^ 20 μL of cell suspension was settled on a Superfrost plus slide (Thermo Fisher Scientific) for 20 min before removing excess solution. Coverslips were carefully placed on top of the cell suspensions. Images were recorded using a 40.0 × 1.10 water objective on a Leica TCS SP5 confocal scanner excited at 514 nm and the images were recorded at 525/600 nm (software: LAS AF 2.7.3). Final images were produced using Fiji (106, 107).

### Amino acid analysis

For analysis of intracellular amino acids of AAT10.2 RNAi cell lines, cells were cultivated in (commercial) SDM79 for 72 h, supplemented or not with 2 mM of each BCAA, in the presence or absence of tetracycline. For amino acid analysis of 3xTy-AAT10.2∗ and 3xTy-Δ35-AAT10.2∗ in the background of the RNAi cell line, tetracycline was applied continuously, *i*.*e*. at least 1 week before the experiment and throughout the experiment, including the washing steps. The cells were cultivated in SDM79S supplemented with 0.4 mM BCAA. Cells were washed twice (800*g* for 5 min) in SDM79S (0 mM BCAA) and further cultivated for 24 h or 72 h in SDM79S supplemented with either 0 or 0.4 mM BCAA. Results are the means of 3 clones (*i*.*e*. biological replicates) of one experiment.

Metabolites were extracted by organic solvent extraction (108). 3 × 10^7^ cells of a mid-log cell cultures were rapidly cooled in ice/water. Cells were washed in ice-cold PBS, and cell pellets were extracted with a mixture of chloroform, methanol, and water at a ratio of 1:3:1, supplemented with 20 μM L-norleucine as an internal standard. Samples were shaken for 1 h at 4 °C. The suspension was centrifuged to remove cell debris, and the clear supernatant was collected and stored until analysis at −80 °C. Free amino acids were determined by ARC (Analytical Research and Services, University of Bern) based on the method of Bidlingmeyer *et al*. (109), therefore, extracts were derivatized with phenyl isothiocyanate and analysis was performed by RP-HPLC coupled to UV detection as described before (28).

Intracellular amino acid amounts are reported as pmol 10^7^ cells^−1^. For conversion into intracellular concentrations, we assumed a cell volume of 3.31 μL per 10^8^ cells (110), an amount of 100 pmol 10^7^ cells^−1^ therefore corresponds to an internal concentration of 302 μM.

### Mass spectrometry analysis

*T. brucei* cells, equivalent to 1 mg of total proteins as determined by Bradford (111) were washed in PBS and frozen at −80 °C. Samples were processed by the Proteomics & Mass Spectrometry Core Facility of the department for BioMedical Research of the University of Bern according to their validated protocols for phospho-proteome analyses (112), and (plasma-)membrane protein enrichment using a two-step ultracentrifugation protocol (113) followed by proteome analysis and data processing as described in the above references, with the exception that MaxQuant version 1.6.14.0 was used and data interpreted against the TriTrypDB-42_TbruceiLister427 and TriTrypDB-50_TbruceiTREU927 protein sequence databases, including some common contaminants. Results are from a single phosphoproteome- and a single (plasma)membrane proteome analysis.

### Comparison of phosphoproteomes

For AATs all phosphosites found in our study were included, but sites with a probability below 95% were marked (Table 1). For comparison of the phosphopeptides found in our study with published phosphoproteome datasets (43, 46, 47), phosphopeptides identified in our study with a phosphorylated site of a probability inferior of 95% were discarded. As published analyses used different *T. brucei* lab strains, each protein identified associated with a phosphorylation site was used to identify the syntenic orthologs in the *T.* *brucei* TREU927 genome sequences (15). Datasets were compared and Venn diagrams designed using Venny 2.1 (114).

### SILAC (stable isotope labeling with amino acids in cell culture)

Cells of the PCF *T. brucei* strain 29-13 were washed in PBS and resuspended in SDM-80 (115) containing 5.55 mM glucose, 10% dialyzed FCS (BioConcept, Switzerland) and either light (^12^C_6_/^14^N_χ_) or heavy (^13^C_6_/^15^N_χ_) isotopes of arginine (1.1 mM) and lysine (0.4 mM) (Euroisotope), as well as 0.4 mM BCAA. The cells were diluted every day to a concentration of 5 × 10^6^ cells mL^−1^ for six days. On day six, the cells were washed twice in SDM-80 containing either 0.04 mM BCAA, 0.4 mM BCAA or 5 mM BCAA and diluted in SDM-80 containing the corresponding BCAA concentration. After 24 h, for the first analysis, 0.75 × 10^8^ cells grown in 0.04 mM BCAA were mixed with 0.75 × 10^8^ cells grown in 0.4 mM BCAA. For the second analysis, 0.75 × 10^8^ cells grown in 0.4 mM BCAA were mixed with 0.75 × 10^8^ cells grown in 5 mM BCAA. The cells were washed in PBS, and the cell pellets were flash-frozen in liquid nitrogen and further processed by the Proteomics & Mass Spectrometry Core Facility of the department for BioMedical Research of the University of Bern, see below. Four biological replicates, including a label switch were prepared for the 0.04/0.4 mM BCAA and 0.4/5 mM BCAA analyses.

Samples were processed and data analyzed by the Proteomics & Mass Spectrometry Core Facility of the Department for BioMedical Research of the University of Bern. For the SILAC-labeled samples, the mass spectrometry data were interpreted with FragPipe (Kong *et al*. 2017) against the TriTrypDB-68_TbruceiTREU927 protein sequences, again including some common contaminants. Search parameters included precursor and fragment mass tolerances of 20 ppm and 0.05 Da; strict trypsin cleavage rule without cuts if proline follows arginine or lysine and allowing two missed cleavages; fixed carbamidomethylation modification on cysteines and oxidation of methionine (maximum 1 time per peptide), protein N-terminal acetylation, phosphorylation of serine/threonine/tyrosine (max. 3x), and heavy label of lysine (+8) and arginine (+10) as variable modifications. Peptide spectrum matches were re-scored with MS-Booster using the Prosit_2019_irt, Prosit_2023_intensity_timsTOF, and DIA-NN models for retention time, fragment spectrum and ion mobility verification, followed by Percolator for false discovery estimation. Peptide intensities and heavy-to-light ratios were calculated with the ionquant algorithm. For the total proteome data, identical search parameters were used with the exception that variable phosphorylation (2x) was only allowed on serine and threonine.

SILAC data analysis: We first filtered the data of contaminants and retained only proteins seen in at least half of the replicates in at least one of the conditions; furthermore, for the phospho-enriched samples, only phosphorylated ions were considered. For both the total proteome and the phospho-enriched samples the log2 ion ratios were first normalized by centering their median to 0 on the respective filtered ion subset. It was noted that a batch effect occurred between the direct ratios and the reversed ratios, so this was corrected using the combat R function from the sva package version 3.59.0 (116). The protein median log2 ratios in the total proteome were then re-calculated as the median of the log2 ratios of contributing ions and used for the calibration of phospho-enriched samples (117). In the latter samples, phospho-peptide and phosphosites log2 ratios were likewise calculated as the median of contributing ions; for those ions allocated to proteins found in the total proteome, calibrated ion ratios could be obtained by dividing them by the corresponding total proteome protein ratio. Differential expression between entities was performed by applying the moderated *t* test after having imputed the missing values by drawing random numbers from a Gaussian distribution of width 0.2x sample standard deviation centered at 0. Multiple testing was corrected using the Benjamini-Hochberg correction.

## Data availability

All data supporting the results of the present study are included in the article. The mass spectrometry proteomics data have been deposited to the ProteomeXchange Consortium *via* the PRIDE (118) partner repository with the dataset identifier PXD079824.

## Supporting information

This article contains supporting information (43, 46, 47).

## Conflict of interest

The authors declare that they have no conflicts of interest with the contents of this article.
