# Supplementary material for: The branched-chain amino acid transporter AAT10.2 of Trypanosoma brucei is regulated by substrate availability
Source: J Biol Chem. 2026 Jul 28;302(9):113379. doi: 10.1016/j.jbc.2026.113379 (PMC13524880; doi:10.1016/j.jbc.2026.113379)
Supplement: Supplementary file 1 [file mmc5.pdf]

Supplementary Figures

The branched-chain amino acid transporter AAT10.2 of *Trypanosoma brucei* is regulated by substrate availability

Quentin-Florian Oliveres, Alexander C. Haindrich, Silvan Piller, Juan P. Macedo, Veerle van Berkum, Fabio Luiz Rogé Ferreira, Christoph Wenger, Arunasalam Naguleswaran, and Doris Rentsch

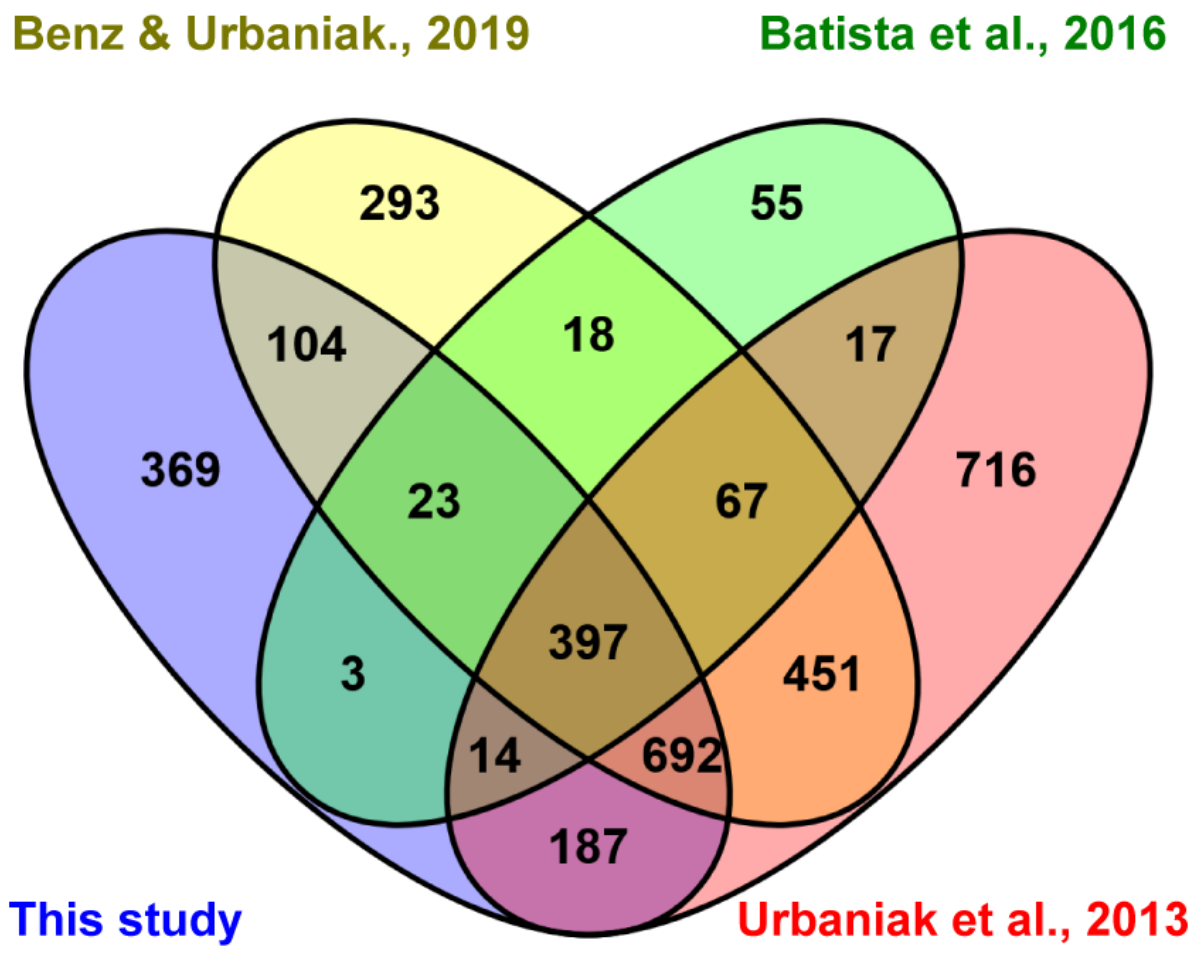

Suppl. Figure 1. Comparison of phosphoproteomes.
Venn diagram shows the overlap of identified phosphorylated proteins between our dataset and phosphorylated proteins published by Batista et al. (47), Benz and Urbaniak (43), and Urbaniak et al. (46).

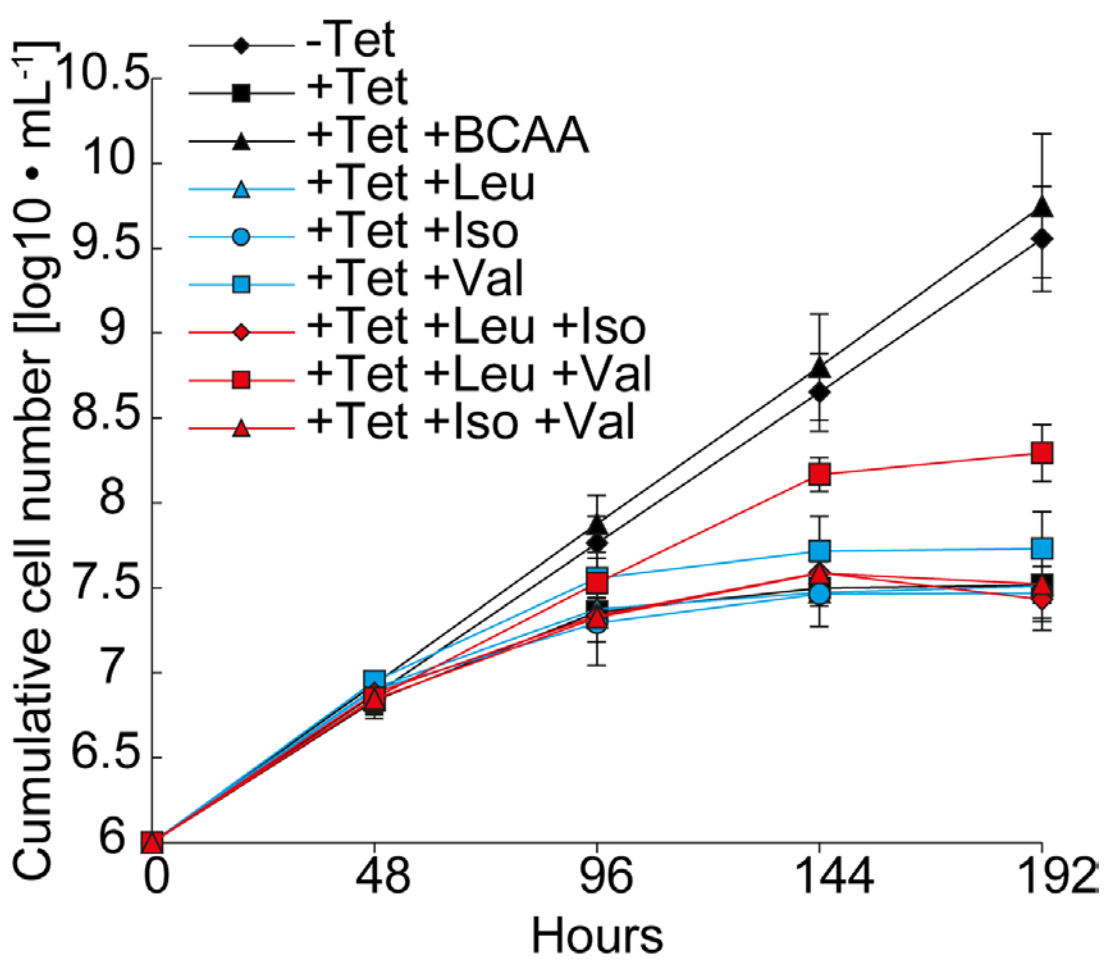

Suppl. Figure 2. Valine, isoleucine and leucine are necessary to rescue the growth arrest observed when down-regulating AAT10.2 by RNAi.

Cumulative growth of AAT10.2 RNAi PCF *T. brucei* cells in SDM79 medium (+/- Tet), supplemented or not with 2 mM valine and/or isoleucine and/or leucine (Val, Iso, Leu) or with all three amino acids (BCAA) (n=3, ±SD).

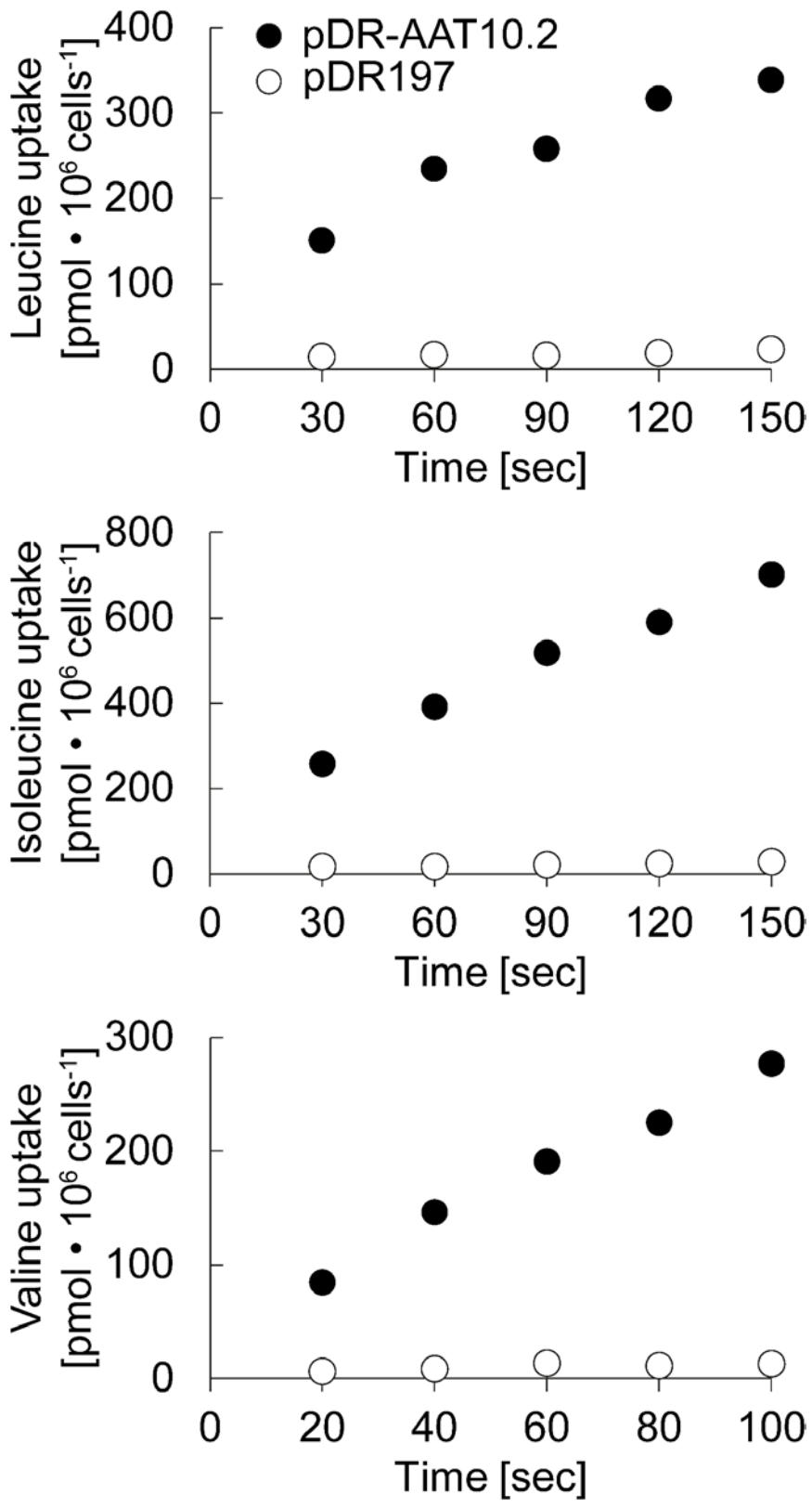

Suppl. Figure 3. AAT10.2 expressed *in S. cerevisiae* supports time-dependent uptake of leucine, valine and isoleucine.

Time-dependent uptake of [^{3}H]-labelled (A) 50 µM L-leucine, (B) 100 µM L-isoleucine, and (C) 50 µM L-valine in *S. cerevisiae* mutant YDR544 expressing AAT10.2 (●) or transformed with vector pDR197 (○). Representative data of one experiment are shown.

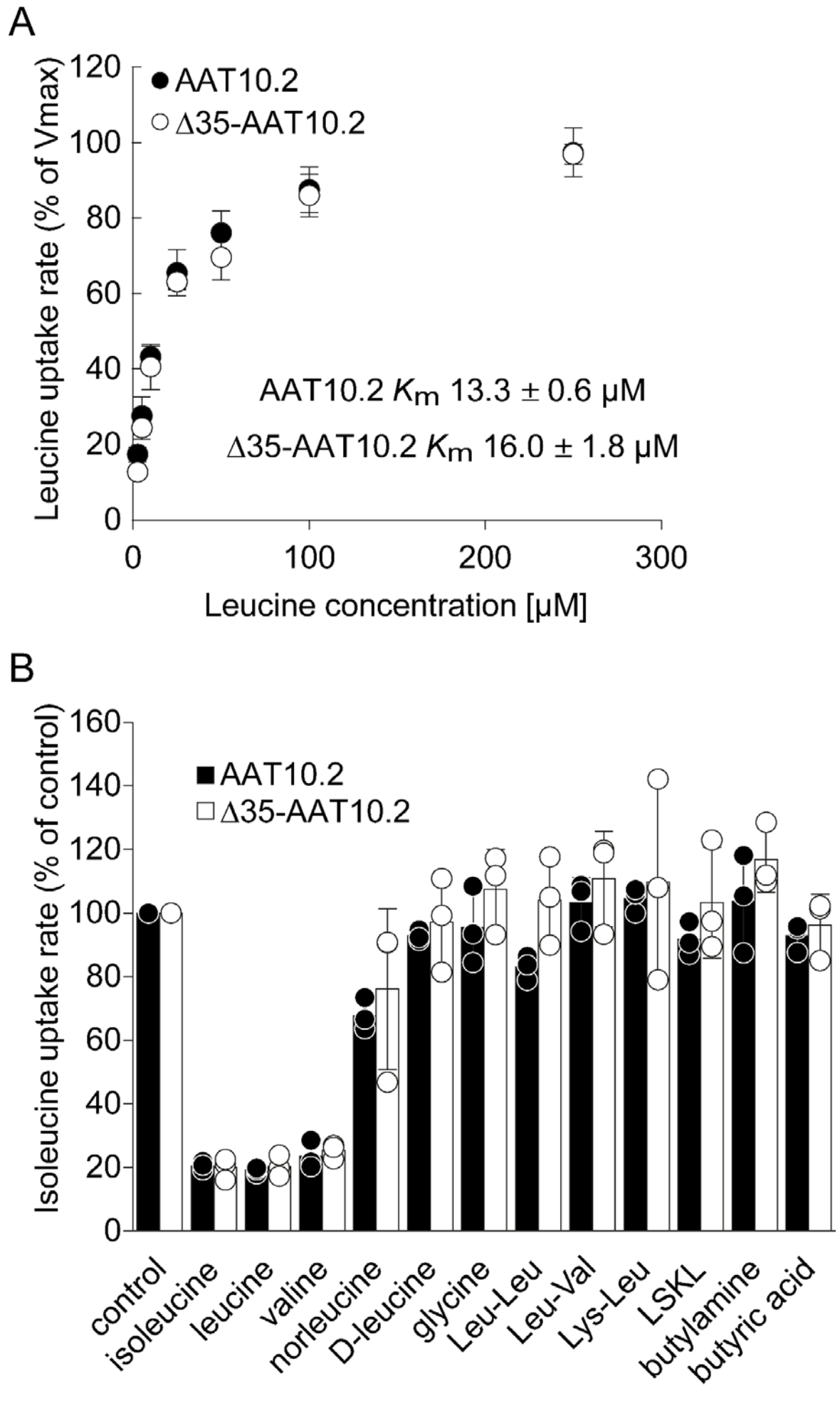

Suppl. Figure 4. Transport properties of AAT10.2 and Δ35-AAT10.2 when expressed in *S. cerevisiae*.

Uptake rates were determined for AAT10.2 and Δ35-AAT10.2 expressed in the *S. cerevisiae* mutant YDR544 as described in Experimental procedures. (A) Kinetics of L-[^{3}H]-leucine transport of AAT10.2 (●) and Δ35-AAT10.2 (○) determined at pH 7.0. Apparent affinities were determined by using the Michaelis-Menten equation (n=3, ±SD). Leucine transport rates at V_{max} were in the range of 79-106 pmol min^{-1} 10^6 cells^{-1}. (B) Uptake rates of 100 μM L-[^{3}H]-isoleucine in the presence of 600 μM competitors. Uptake rates were normalized to the control with no competitor added – set to 100% (n=3, ±SD). Dots show individual data points. LSKL, Leu-Ser-Lys-Leu.

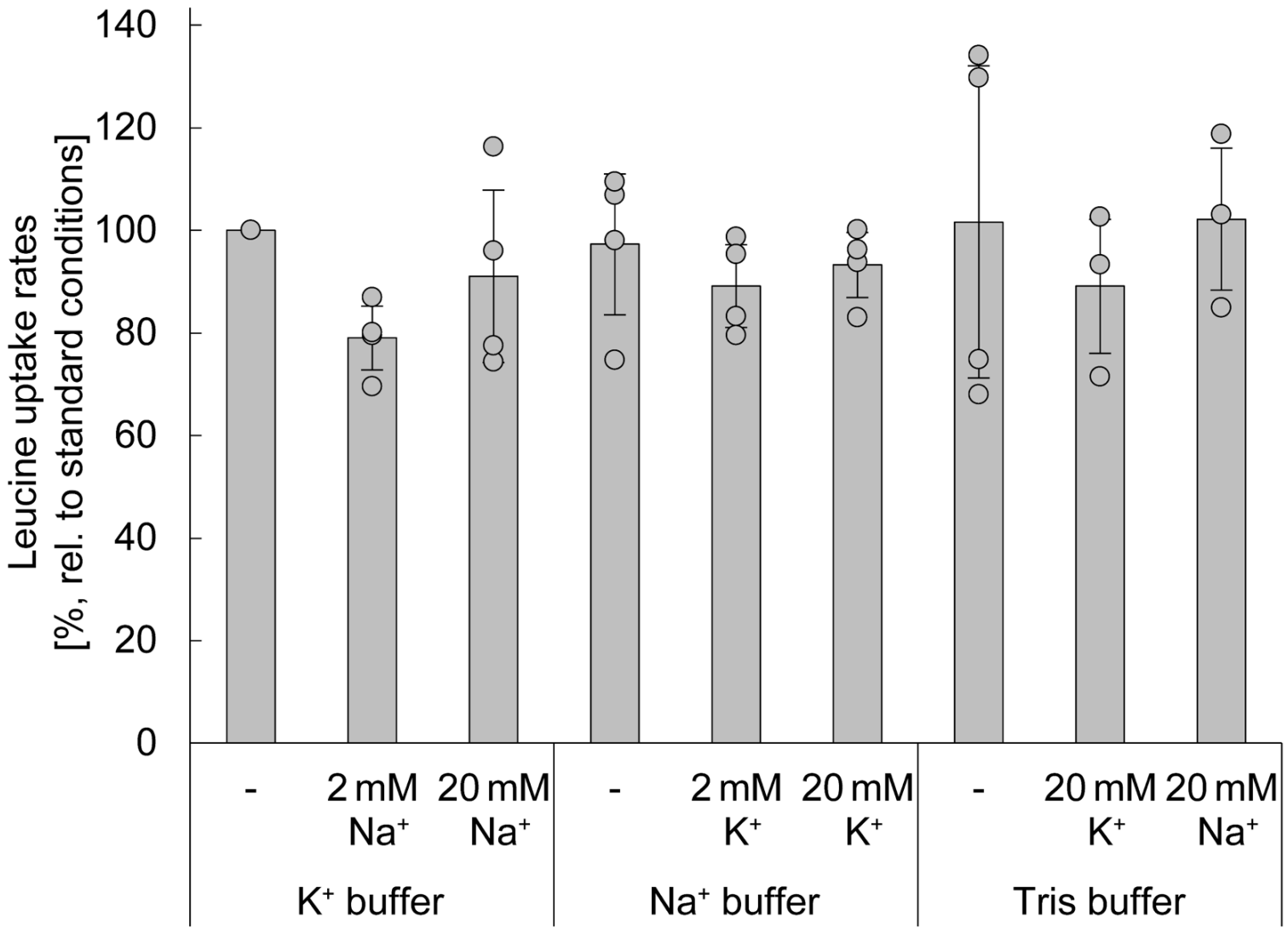

Suppl. Figure 5. Leucine transport by AAT10.2 in *S. cerevisiae* is not increased by Na^+ or K^+.

Uptake rates of 200 µM L-[^{3}H]-leucine were determined for AAT10.2 expressed in the *S. cerevisiae* mutant YDR544 as described in Experimental procedures. The buffer was lacking either Na^+ (50 mM potassium phosphate), K^+ (50 mM sodium phosphate), or both ions (50 mM TRIS-phosphate (pH 7), and was supplemented with 0, 2, or 20 mM NaCl or KCl. Uptake rates were normalized on the uptake rates in potassium phosphate buffer without sodium (pH 7) - set to 100% (n=3-4, ±SD). Dots show individual data points. Leucine transport rates were in the range of 642 to 1555 pmol min^{-1} 10^8 $cells^{-1}$.

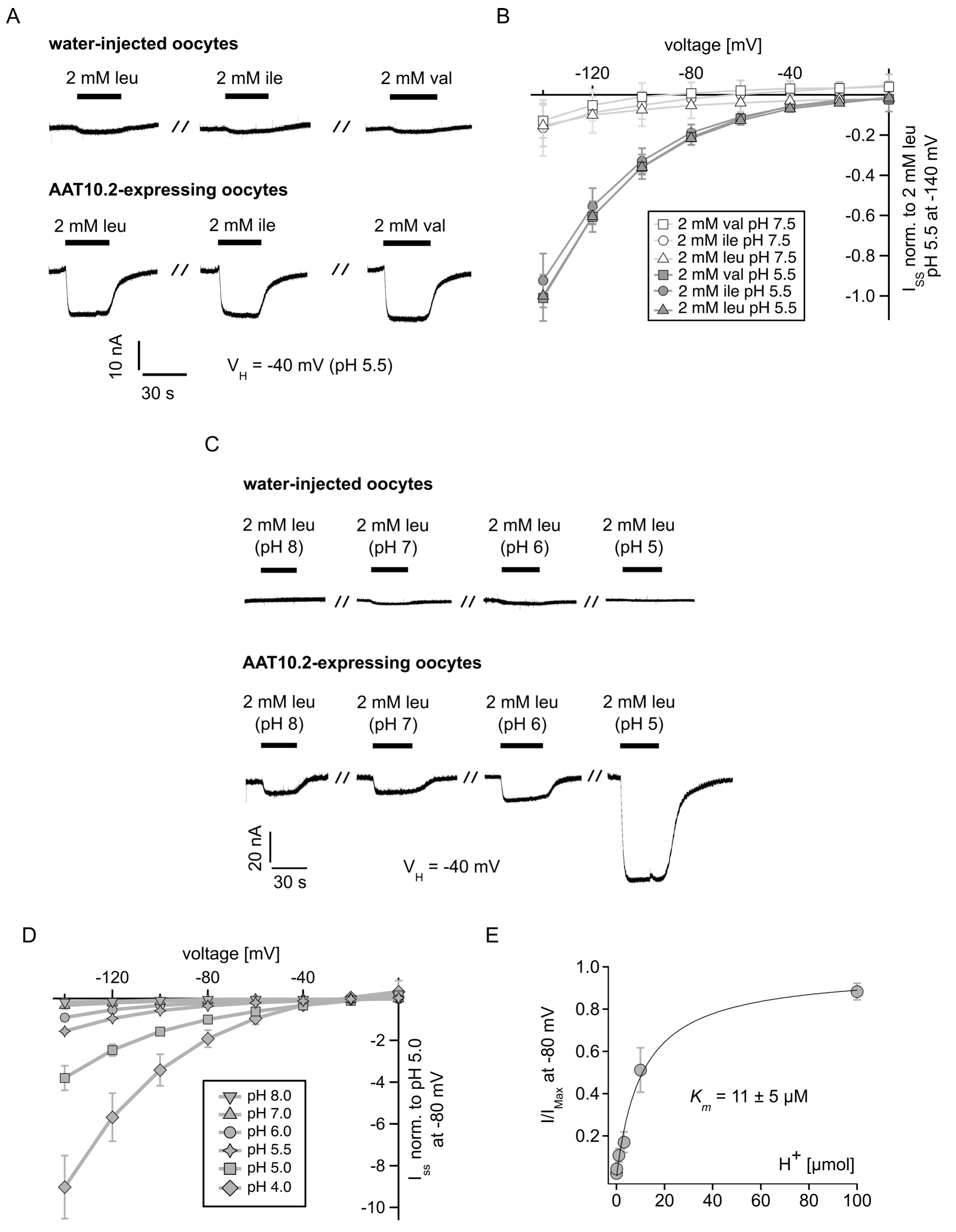

Suppl. Figure 6. Substrate-induced currents in oocytes expressing AAT10.2.
(A) Current traces recorded at V_m of -40 mV and pH 5.5 upon addition of 2 mM of leucine, isoleucine or valine in oocytes expressing AAT10.2 and water-injected oocytes. (B) Voltage-dependence of currents induced by 2 mM leucine, isoleucine or valine at pH 7.5 and 5.5. Steady-state substrate-induced currents (I_{ss}) were normalized to currents induced by 2 mM leucine at -140 mV, pH 5.5 (n=8, ± SD). (C) 2 mM leucine induced currents in water-injected oocytes or oocytes expressing AAT10.2 at the pH values indicated (V_m -40 mV). (D) Voltage-dependence of 2 mM leucine induced currents at different pH. Currents were normalized to substrate-induced currents at pH 5 and V_m -80 mV (n=8; ±SD). (E) Proton dependence of 2 mM leucine transport is shown for V_m -80 mV. Steady-state currents were normalized on I_{max} (data of (D)). All experiments were performed with oocytes from at least two batches.

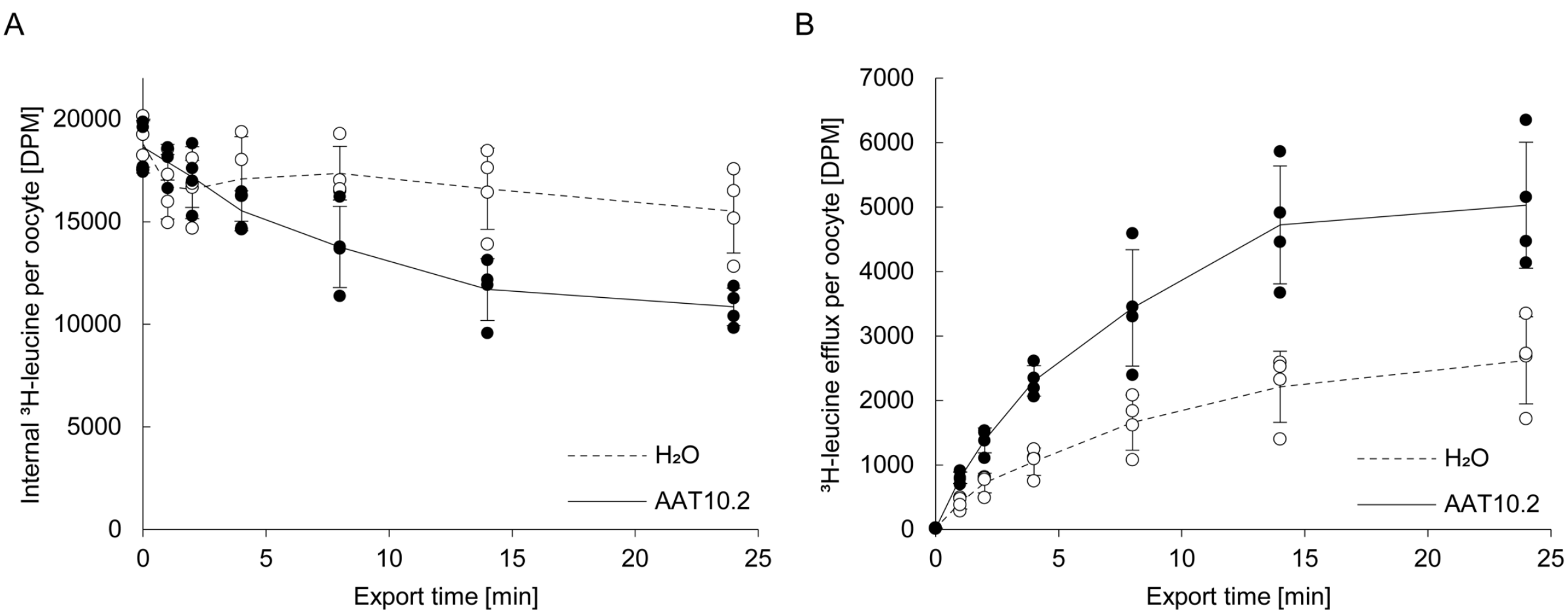

Suppl. Figure 7. Leucine efflux from oocytes expressing AAT10.2.
Efflux was measured using oocytes injected with water (○, dashed lines, H_2O) or expressing AAT10.2 (●, full lines) by injecting 10 pmol L-[^{3}H]-leucine and measuring (A) the remaining radioactivity in the oocytes at the time points indicated (n=4, ±SD), and (B) accumulation of radioactivity released into the export buffer from the oocytes measured in (A). Dots show individual data points, which is in (A) the average of 3 oocytes. Experiments were performed with two different batches of oocytes.

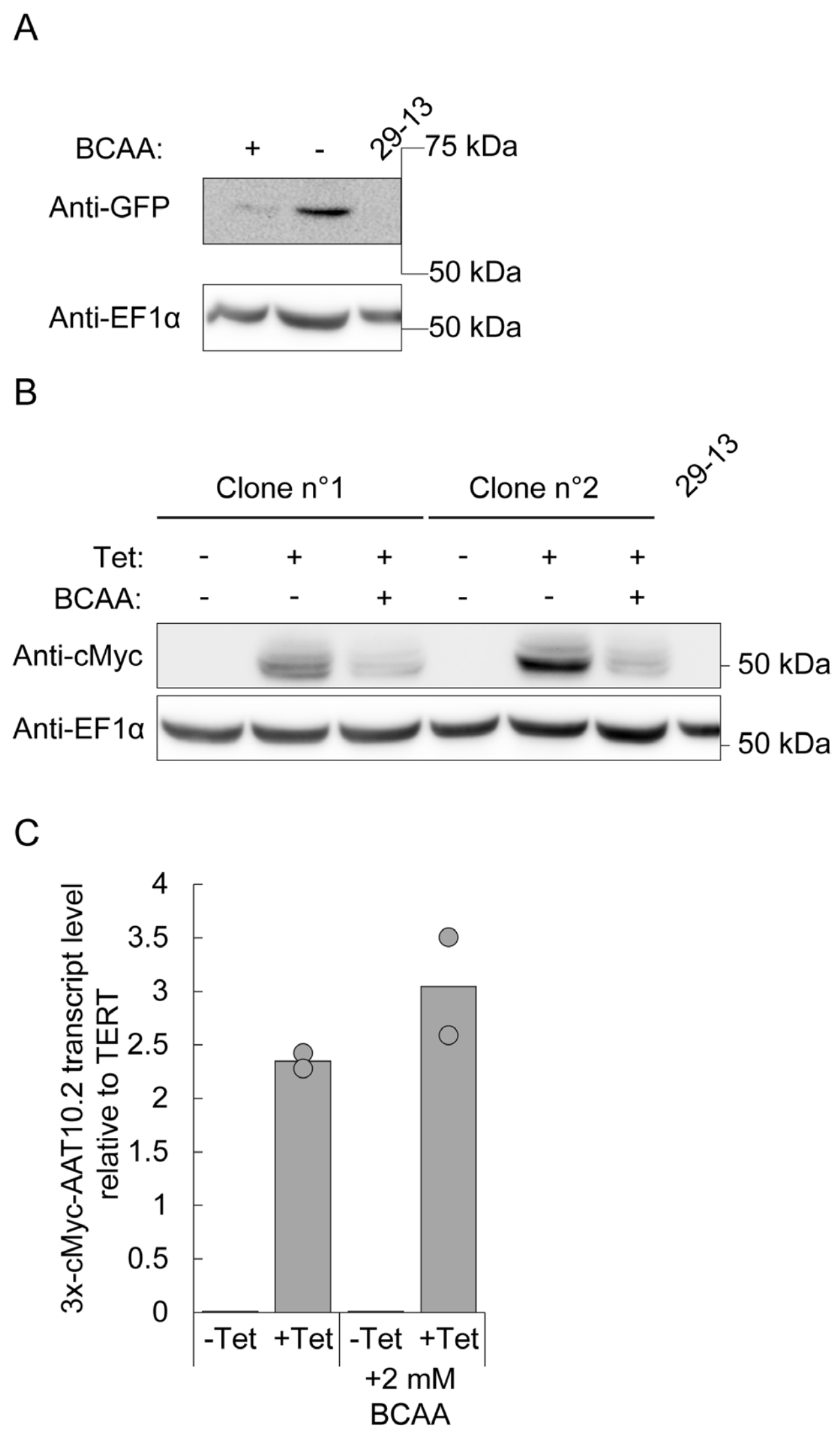

Suppl. Figure 8. eYFP-AAT10.2 and 3xcMyc-AAT10.2 are sensitive to the BCAA concentrations in the medium.

(A) Immunoblot analyses of *in situ*-tagged eYFP-AAT10.2 cultivated in SDM79 supplemented or not with 2 mM BCAA. As control the parental cell line 29-13 is shown. Detection of eYFP using a GFP-antibody. EF-1α was used as control. $5x10^6$ cells per sample; (n=1).

(B, C) *T. brucei* ectopically expressing 3xcMyc-AAT10.2 under control of a tetracycline-inducible promoter. Cells were induced (+Tet) or not (-Tet) with tetracycline and cultured for 24 h in SDM79 supplemented or not with 2 mM BCAA. (B) Immunoblot analysis using an anti-cMyc antibody (two different clones). EF-1α was used as control. (C) 3xcMyc-AAT10.2 transcript levels determined by qRT-PCR, from clone n°1, using one (-Tet) or two (+Tet) replicates. qRT-PCR was performed using QO71 and QO72 primers, specific for the cMyc-tagged AAT10.2 transcript and compared to TERT. qPCR was performed with three different cDNA dilutions (1:40, 1:120 and 1:360), each with two technical replicates, average values of the 6 values were used as one data point.

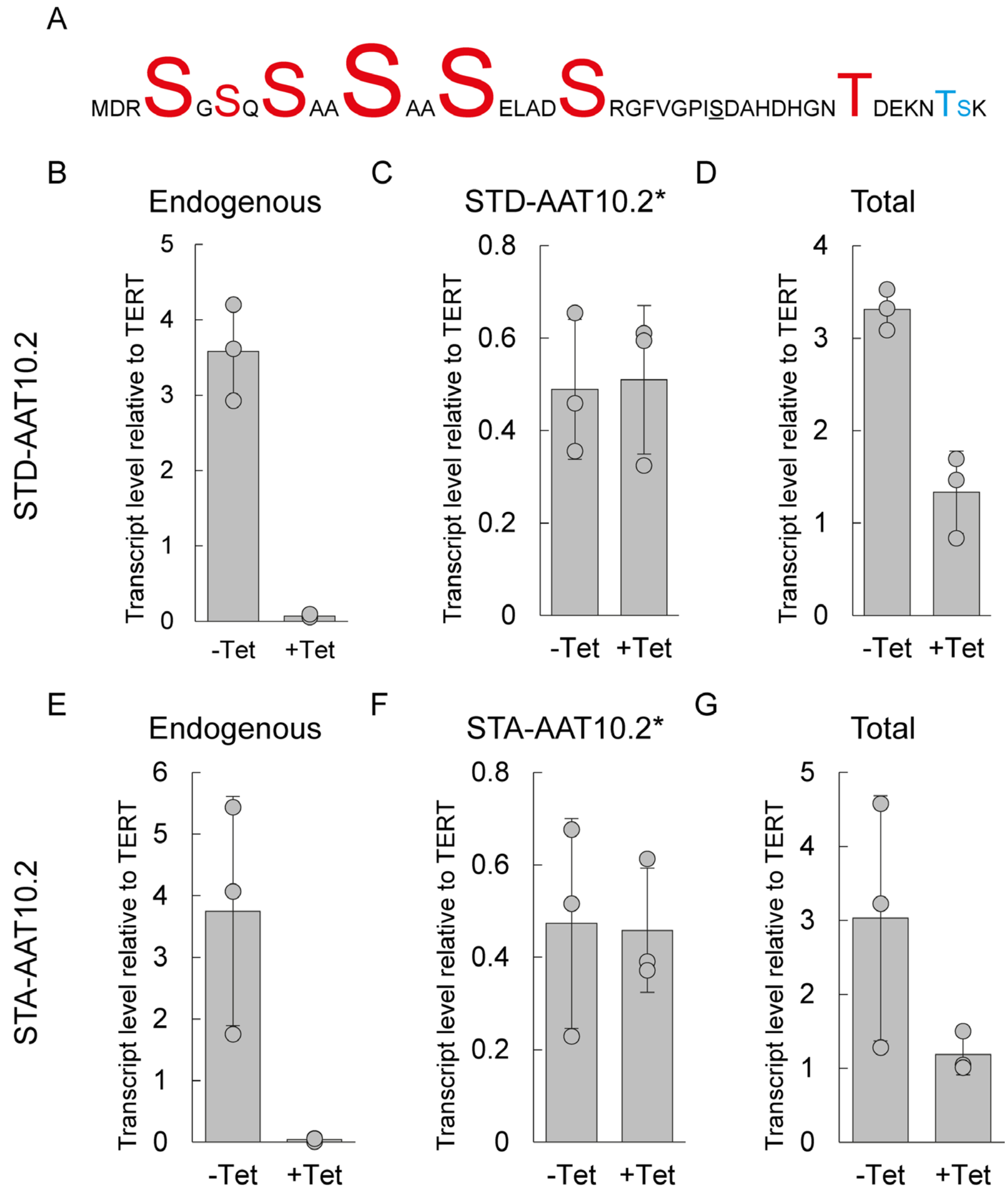

Suppl. Figure 9. mRNA levels of endogenous AAT10.2, phosphomimicking mutant 3xTy-STD-AAT10.2* and non-phosphorylatable 3xTy-STA-AAT10.2*.

(A) Phosphorylated residues of AAT10.2 as identified in the proteome analysis (Suppl. Table 2). Red, phosphorylated residues (localization probability ≥0.95); underlined, S27 was not found phosphorylated in our study, but described to be phosphorylated by Benz and Urbaniak (43); blue, T40 and S41 were detected with a probability below 95% in our study, but were previously reported to be phosphorylated (43, 47). Size of letters represents the probability score.

(B-G) AAT10.2 mRNA levels in AAT10.2 RNAi PCF *T. brucei* cultured for 72 h in SDM79 and expressing 3xTy-STD-AAT10.2* (B, C and D) or 3xTy-STA-AAT10.2* (E, F and G) in the presence or absence of tetracycline (+/- Tet) to induce RNAi. mRNA levels of (B and E) endogenous AAT10.2, (C) 3xTy-STD-AAT10.2*, (F) 3xTy-STA-AAT10.2*, and (D and G) total AAT10.2 mRNA (i.e. codon altered AAT10.2* plus endogenous AAT10.2) normalized to TERT (n=3, ± SD).

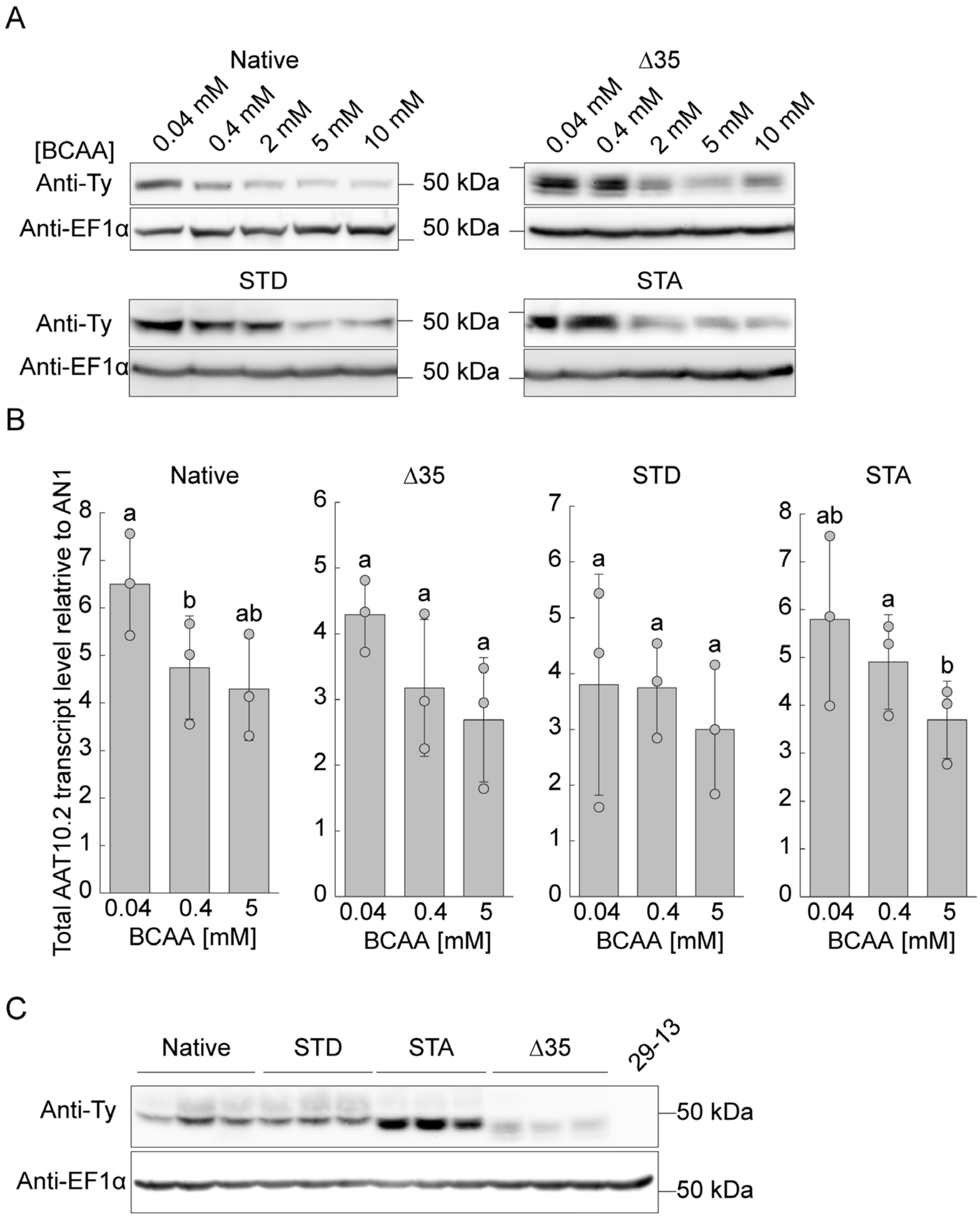

Suppl. Figure 10. AAT10.2 protein and mRNA are dependent on the BCAA concentration in the medium and the N-terminal hydrophilic region of AAT10.2.
(A) Immunoblot analysis using induced AAT10.2 RNAi lines expressing 3xTy-AAT10.2* (native), 3xTy-Δ35-AAT10.2* (Δ35), 3xTy-STD-AAT10.2* (STD), or 3xTy-STA-AAT10.2* (STA) 24 h after changing the BCAA concentration in medium SDM79S from 0.4 mM to 0.04, 0.4, 2, 5 or 10 mM BCAA. EF-1α was used as control. (B) Associated total AAT10.2 mRNA levels (i.e. codon altered AAT10.2* plus endogenous AAT10.2) at 0.04, 0.4, and 5 mM BCAA relative to AN1 (n=3, ±SD). Please note that RNA of the different variants was analyzed in different batches, therefore RNA levels can only be compared within one variant (i.e. depending on the BCAA levels), and not between variants. (C) Immunoblot analysis of 3xTy-AAT10.2* variants expressed in the induced AAT10.2 RNAi line; i.e. 3xTy-AAT10.2* (native), 3xTy-Δ35-AAT10.2* (Δ35), phosphomimicking mutant (STD), and non-phosphorylatable mutant (STA). Cells were cultured in SDM79. For each variant 3 different clones ($5x10^6$ cells) were used. Results shown in (C) are not from the same experiment as those shown in (A) and (B). Protein was detected using anti-Ty or anti-EF-1α antibodies.

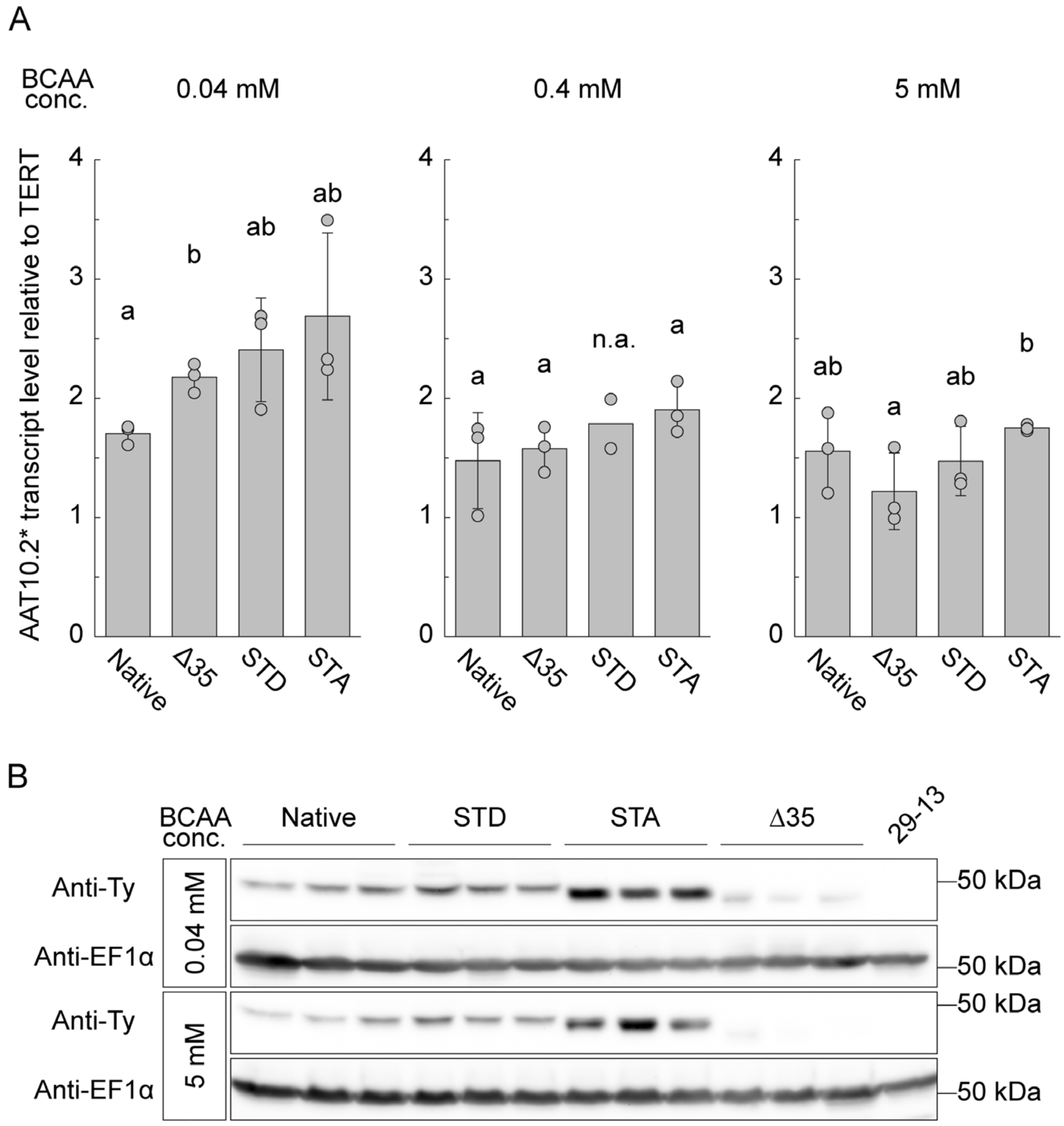

Suppl. Figure 11. AAT10.2 mRNA levels are comparable between variants while protein abundance varies.

(A) mRNA levels of codon altered AAT10.2* at 0.04, 0.4, and 5 mM BCAA relative to TERT . Data used is the same as in Figure 5B. Compact letters indicate statistically significant differences between variants, error bar represent standard deviation (p-value<0.05, two-tailed t-test, n=3); for STD at 0.4 mM BCAA n=2, n.a. not analyzed). (B) Immunoblot analysis of 3xTy-AAT10.2* variants expressed in the induced AAT10.2 RNAi line; i.e. 3xTy-AAT10.2* (native), 3xTy-Δ35-AAT10.2* (Δ35), phosphomimicking mutant (STD), and non-phosphorylatable mutant (STA), 24 h after changing the BCAA concentration in medium SDM79S from 0.4 mM to 0.04 mM or 5 mM BCAA. For each variant 3 different clones ($5x10^6$ cells) were used. Cells are from the same experiment as in Figure 5. Protein was detected using anti-Ty or anti-EF-1a antibodies.

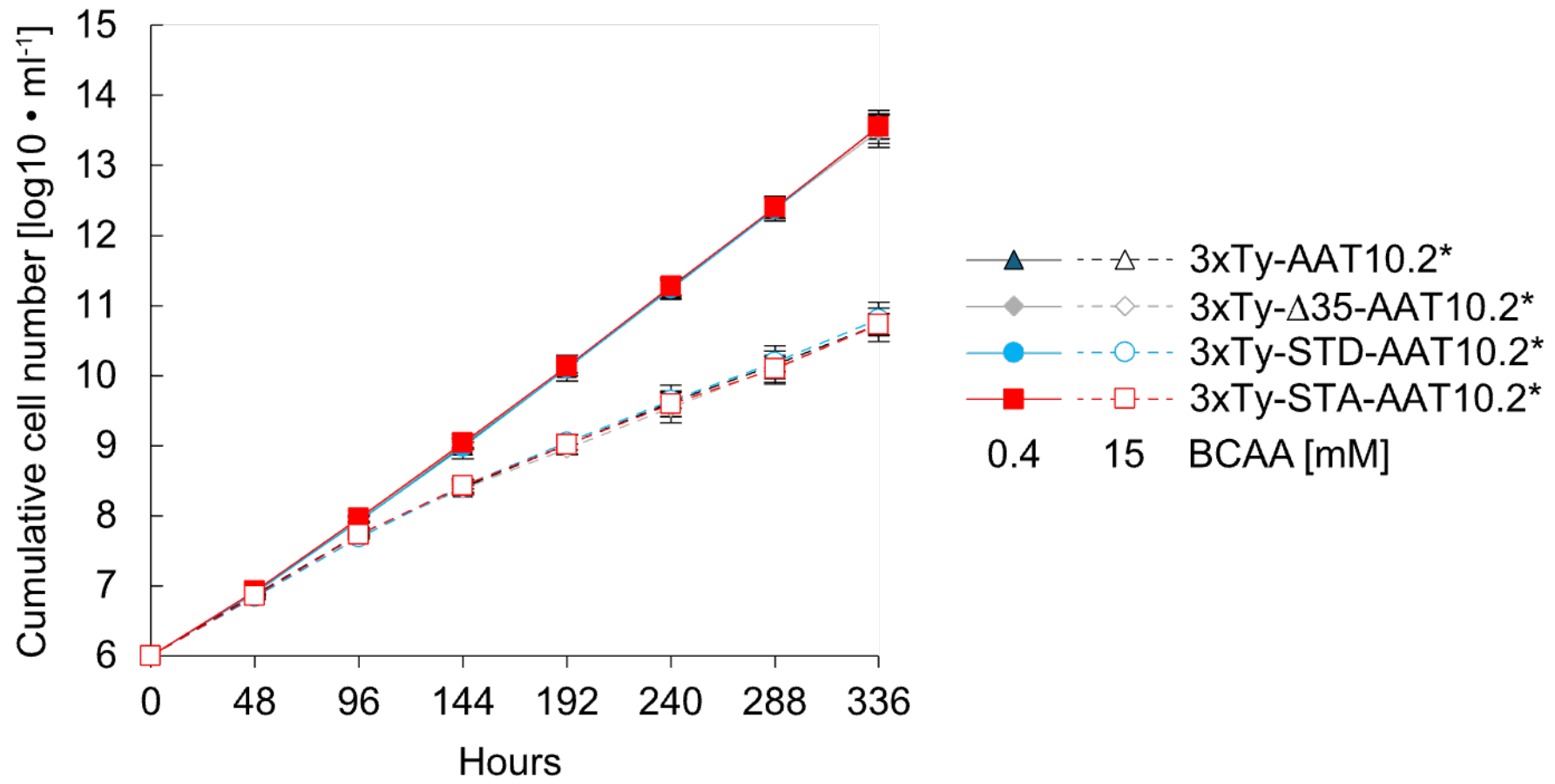

Suppl. Figure 12. Cell growth at high BCAA availability is comparable among variants.

Cumulative cell growth of induced AAT10.2 RNAi lines expressing 3xTy-AAT10.2*, 3xTy-Δ35-AAT10.2*, 3xTy-STA-AAT10.2*, or 3xTy-STD-AAT10.2*, cultured in SDM79S and supplemented either with 0.4 mM (solid lines and full symbols) or 15 mM BCAA (dashed lines and empty symbols) (n=3, ±SD).

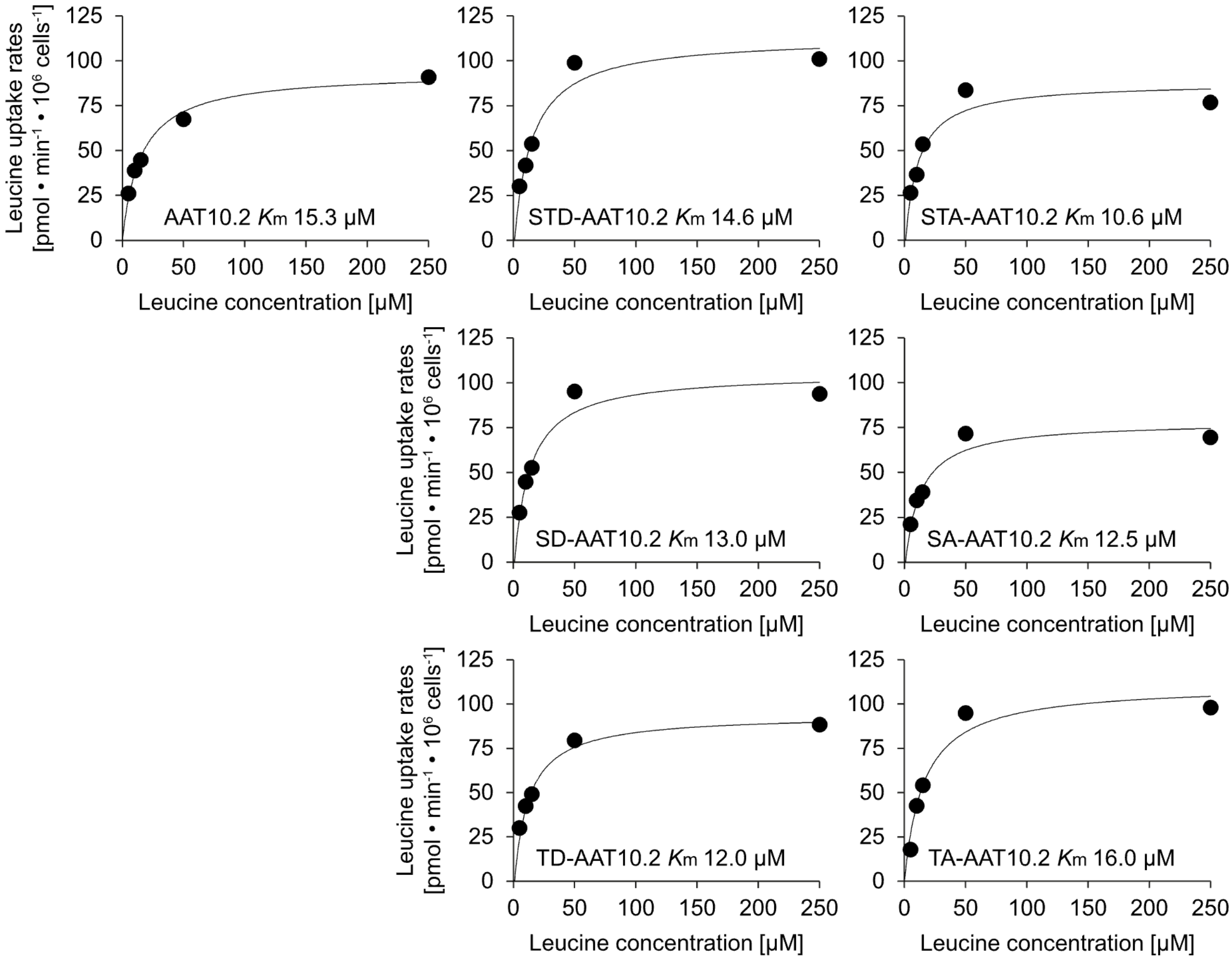

Suppl. Figure 13. Kinetics of AAT10.2 phosphomimicking mutants remain comparable to wildtype AAT10.2.
[^{3}H]-L-leucine transport kinetics of AAT10.2 or mutated AAT10.2 versions expressed in *S. cerevisiae* mutant YDR544 at pH 7.0. Uptake rates were calculated as described in Experimental procedures. Nomenclature of the mutant: T35A (TA-AAT10.2); T35D (TD-AAT10.2); S4A, S6A, S8A, S11A, S14A, S19A (SA-AAT10.2); S4D, S6D, S8D, S11D, S14D, S19D (SD-AAT10.2). STA-AAT10.2 corresponds to the mutated version of AAT10.2 carrying all mutations of TA and SA, similarly STD-AAT10.2 contains all TD and SD mutations (n=1).

A

B

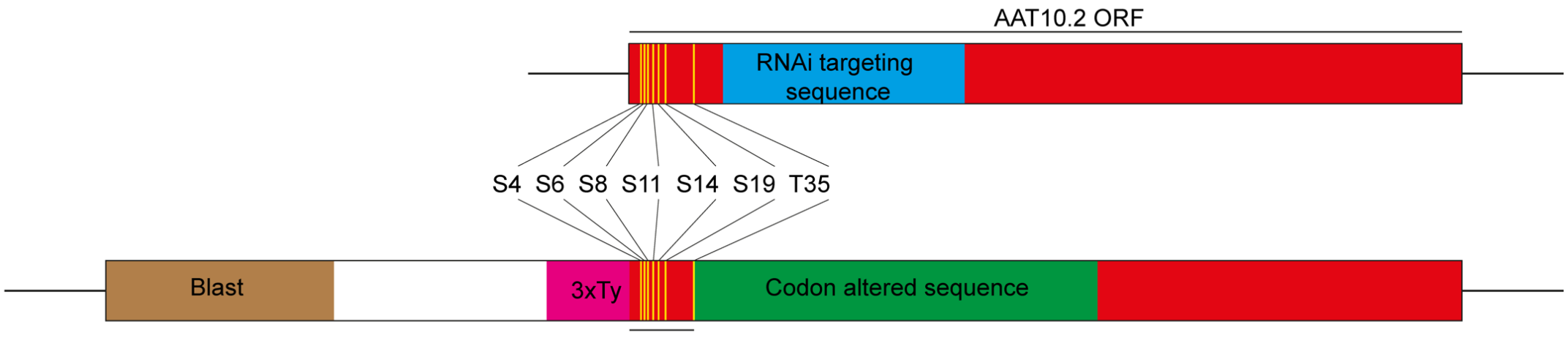

C

```
>Nucleotide Alignment of the first 79bp of AAT10.2 and AAT10.2*
AAT10.2 ORF (1-790)          1 ATGGACAGATCTGGCAGTCAATCCGCAGCATCAGCGGCGTCGGAACTTGCTGACAGTCGG   60
Consensus                        ATGGACAGATCTGGCAGTCAATCCGCAGCATCAGCGGCGTCGGAACTTGCTGACAGTCGG
pUC AAT10.2^790-CA           1 ATGGACAGATCTGGCAGTCAATCCGCAGCATCAGCGGCGTCGGAACTTGCTGACAGTCGG   60

AAT10.2 ORF (1-790)         61 GGTTTTGTGGGGCCAATATCGGATGCCCATGACCACGGAAATACTGACGAGAAAAATACC  120
Consensus                        GGTTTTGTGGGGCCAATATCGGATGCCCATGACCACGGAAATACTGACGAGAAAAA-ACC
pUC AAT10.2^790-CA          61 GGTTTTGTGGGGCCAATATCGGATGCCCATGACCACGGAAATACTGACGAGAAAAACACC  120

AAT10.2 ORF (1-790)        121 TCAAAAGCAAAGGATTCAAATGGTTTCTTCTCGAAGGTGAGTTTATGCATCGCCACTGTT  180
Consensus                        TCAAAAGCAAA-GATTCAAATGGTTT-TT-TCGAA-GT-----T-TG-AT-GC-AC-GT-
pUC-AAT10.2^790-CA         121 TCAAAAGCAAAAGATTCAAATGGTTTTTTTTCGAAAGTTTCACTCTGTATTGCGACCGTG  180

AAT10.2 ORF (1-790)        181 TTACCTCCAGGCGGCATTGCGGCGAGTGCTTTCAACATGGCCTCAACAACTCTTGGCGGC  240
Consensus                        -T-CC-CC-GG-GG-AT-GC-GC---TGC-TT-AA-ATGGC-TC-AC-AC--T-GG-GG-
pUC-AAT10.2^790-CA         181 CTCCCACCGGGAGGAATCGCTGCTTCTGCATTTAATATGGCTTCCACGACCTTAGGTGGT  240

AAT10.2 ORF (1-790)        241 GGTATTATTGGGATGCCAGCAGCAACCAACAGCAGTGGACTTGTTATGGGATTATTTTAT  300
Consensus                        GG-AT-AT-GG-ATGCC-GC-GC-AC-AA---C---GG--T-GT-ATGGG--T-TT-TA-
pUC-AAT10.2^790-CA         241 GGCATCATCGGCATGCCTGCGGCGACTAATTCCTCCGGGTTAGTGATGGGGCTCTTCTAC  300

AAT10.2 ORF (1-790)        301 CTCATGCTCATTTCCTCCGTAACCGTTTTTACAATGCACAACCTTTCCATTGCAGCGGAG  360
Consensus                        CT-ATGCT-AT-TC-TC-GT-AC-GT-TT-AC-ATGCA-AA--T----AT-GC-GC-GA-
pUC-AAT10.2^790-CA         301 CTTATGCTTATATCTTCTGTTACTGTGTTCACCATGCATAATTTAAGTATCGCCGCAGAA  360

AAT10.2 ORF (1-790)        361 CGCACCAACACCCACACGTTTGAGGAGGTGACTCGTGTGCTTCTTGGCCGTGGGGCTGCA  420
Consensus                        CG-AC-AA-AC-CA-AC-TT-GA-GA-GT-AC-CG-GT--T--T-GG-CG-GG-GC-GC-
pUC-AAT10.2^790-CA         361 CGTACTAATACTCATACATTCGAAGAAGTTACACGCGTTTTGTTGGGGCGCGGCGCAGCC  420

AAT10.2 ORF (1-790)        421 TATATTCTCGCTGCTATCCGAGCATTCCTTGGGTTTAGTGCCTGTGTTGCTTTTGTTATA  480
Consensus                        TA-AT-CT-GC-GC-AT-CG-GC-TT-CT-GG-TT----GC-TG-GT-GC-TT-GT-AT-
pUC-AAT10.2^790-CA         421 TACATCCTTGCAGCAATTCGTGCCTTTCTAGGCTTCTCAGCTTGCGTCGCATTCGTAATT  480

AAT10.2 ORF (1-790)        481 TCTTTGGGGGATATTATGTCTTCTATTCTAAATGGCACTAATGCACCGGATTTTTGGAAA  540
Consensus                        --T-T-GG-GA-AT-ATG--T---AT-CT-AA-GG-AC-AA-GC-CC-GA-TT-TGGAA-
pUC-AAT10.2^790-CA         481 AGTCTTGGCGACATCATGAGTAGCATCCTTAACGGGACCAACGCTCCAGACTTCTGGAAG  540

AAT10.2 ORF (1-790)        541 GAAAAATCAGGTAACCGCGTCTTGACGGTAATAGTTTGGGCATGTTGTATGCTTCCACTT  600
Consensus                        GA-AA----GG-AA-CG-GT--T-ACGGTAATAGTTTGGGCATGTTGTATGCTTCCACTT
pUC-AAT10.2^790-CA         541 GAGAAGAGTGGCAATCGAGTTCTTACGGTAATAGTTTGGGCATGTTGTATGCTTCCACTT  600

AAT10.2 ORF (1-790)        601 GTGATACCGCGACATGTGGATTCACTTCGTCATGTTTCCACCTGTGCCGTTACTTTCATG  660
Consensus                        GTGATACCGCGACA-GTGGATTCACTTCGTCATGTTTCCACCTGTGCCGTTACTTT-ATG
pUC-AAT10.2^790-CA         601 GTGATACCGCGACACGTGGATTCACTTCGTCATGTTTCCACCTGTGCCGTTACTTTTATG  660

AAT10.2 ORF (1-790)        661 GTGTATTTTGTCATTGTGATTGTTGTGCACTCATGTCTAAATGGGCTTCCGGAGAATATT  720
                                 GTGTATTTTGTCATTGTGATTGTTGTGCACTCATGTCTAAA-GGGCTTCCGGAGAA-ATT
pUC-AAT10.2^790-CA         661 GTGTATTTTGTCATTGTGATTGTTGTGCACTCATGTCTAAACGGGCTTCCGGAGAACATT  720

AAT10.2 ORF (1-790)        721 AAGAGCGTTAGTGTTGGAAAGAGTGATACAGCTGAAATTATCCTTTTCAACACCGGAAAC  780
                                 AAGAGCGTTAGTGTTGGAAAGAGTGATACAGCTGA-ATTAT-CTTTT-AACACCGGAAAC
pUC-AAT10.2^790-CA         721 AAGAGCGTTAGTGTTGGAAAGAGTGATACAGCTGAGATTATTCTTTTTAACACCGGAAAC  780

AAT10.2 ORF (1-790)        781 AAGGCAATTG  790
                                 AAGGCAATTG
pUC-AAT10.2^790-CA         781 AAGGCAATTG  790
```

Suppl. Figure 14. Schematic representation of AAT10.2 and codon-altered variant as well as corresponding sequences.

(A) Wild type AAT10.2 locus. (B) 3xTy-AAT10.2* used for replacement (cloned in pPOTv6-Blast). (C) DNA sequence of the first 790 bp of AAT10.2 aligned with codon altered AAT10.2* (pUC AAT10.2$^{790\text{ -CA}}$) and the consensus sequence. Dashes (-) in the consensus sequence indicate differences between the AAT10.2 and AAT10.2* sequences. Red, the AAT10.2 gene; blue, the region targeted by the AAT10.2 RNAi construct; yellow, the codons of the phosphorylated residues that were altered; green, the region with altered codon usage, underlined, the region truncated for Δ35-AAT10.2; Blast, blasticidin resistance marker.

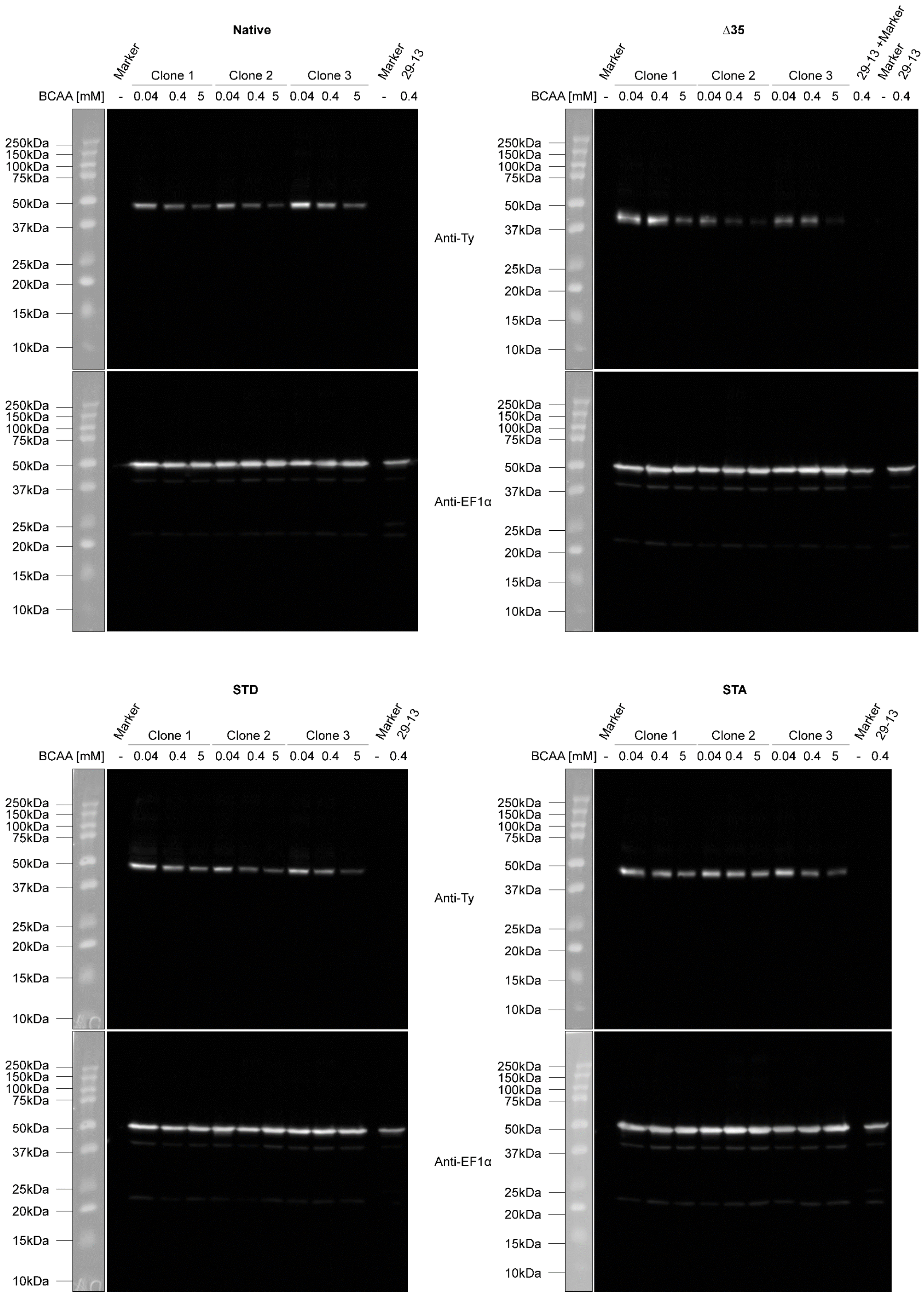

Suppl. Figure 15. Unedited full blots of Figure 5A.

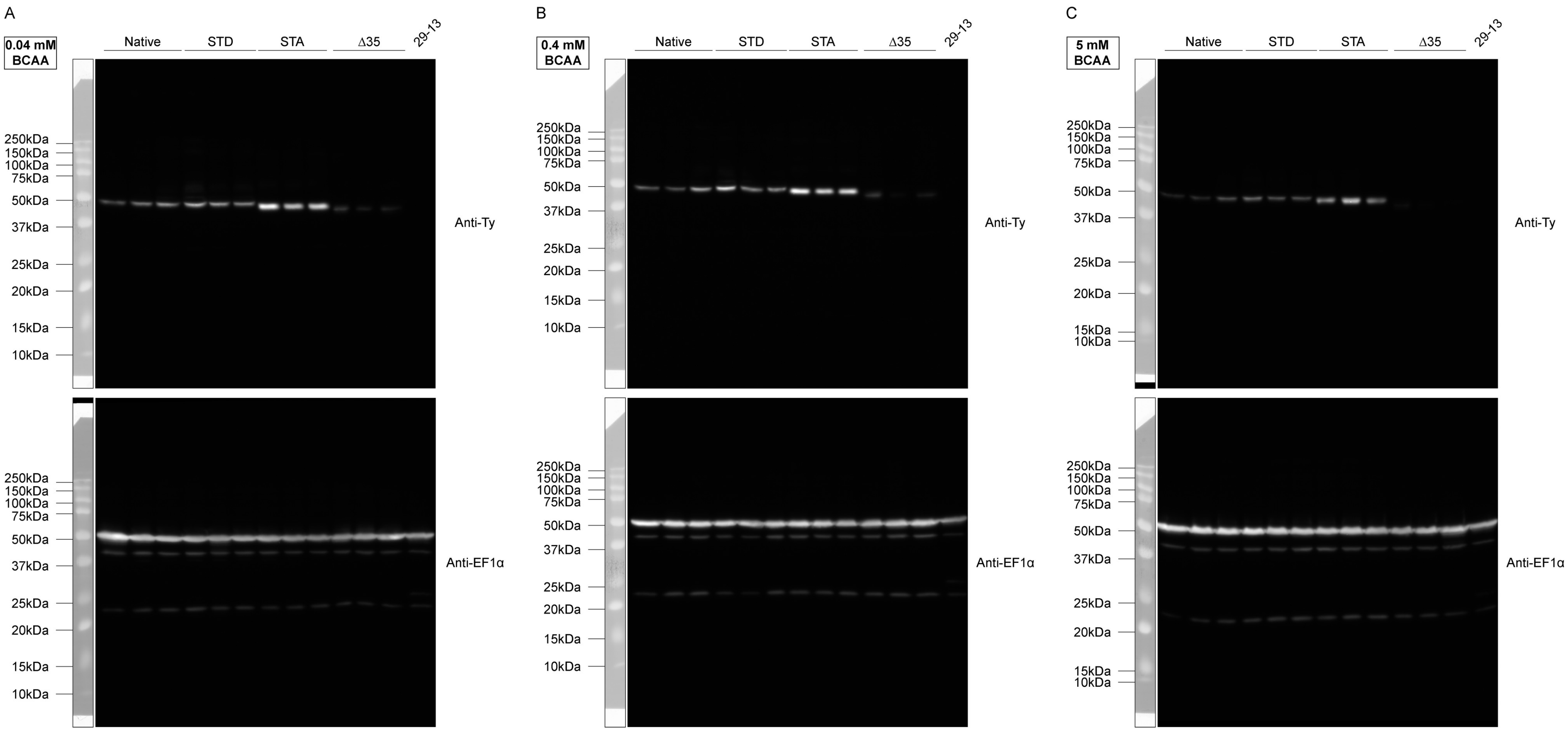

Suppl. Figure 16. Unedited full blots of Figure 5C and Supplementary Figure 11B.

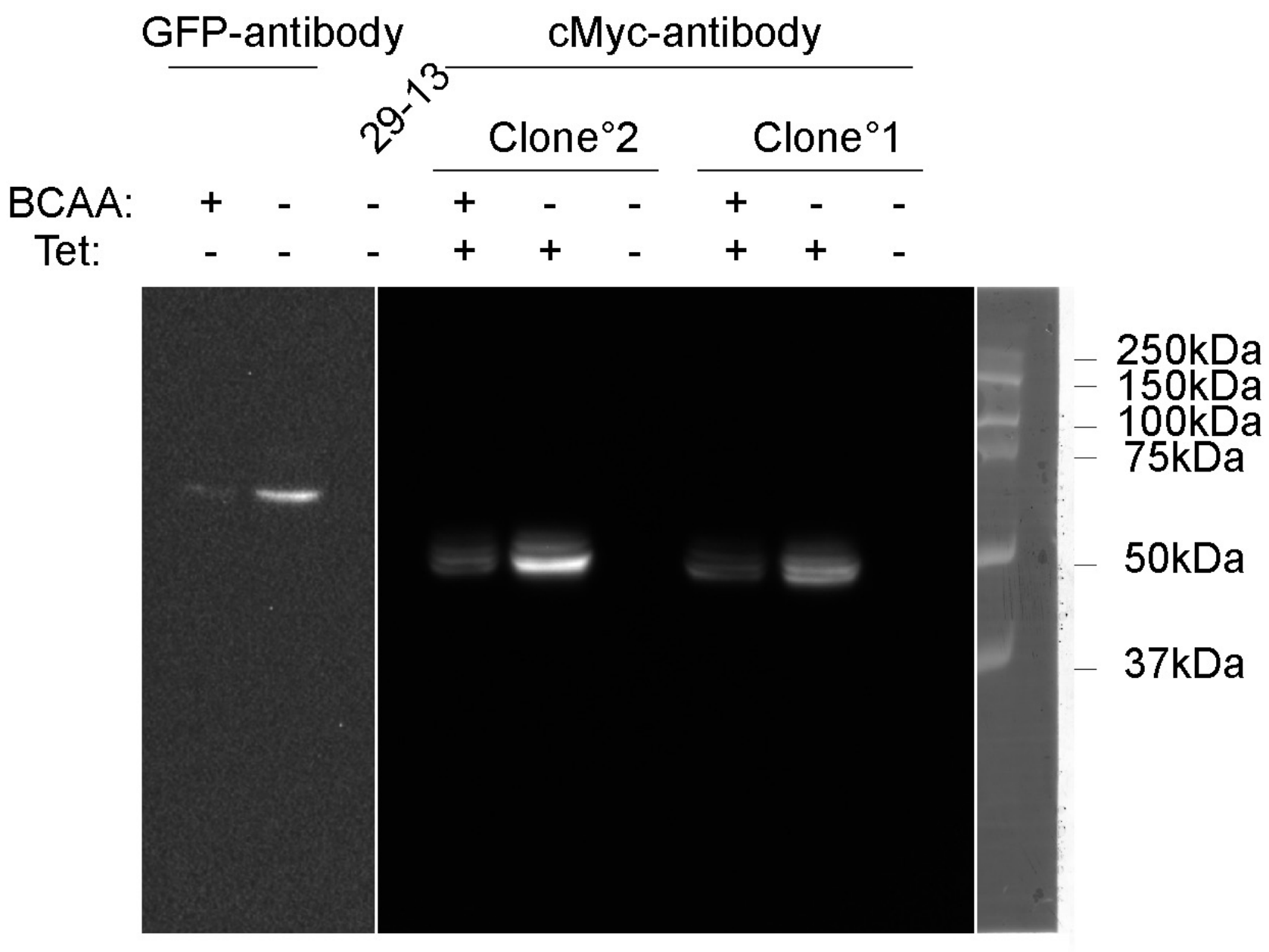

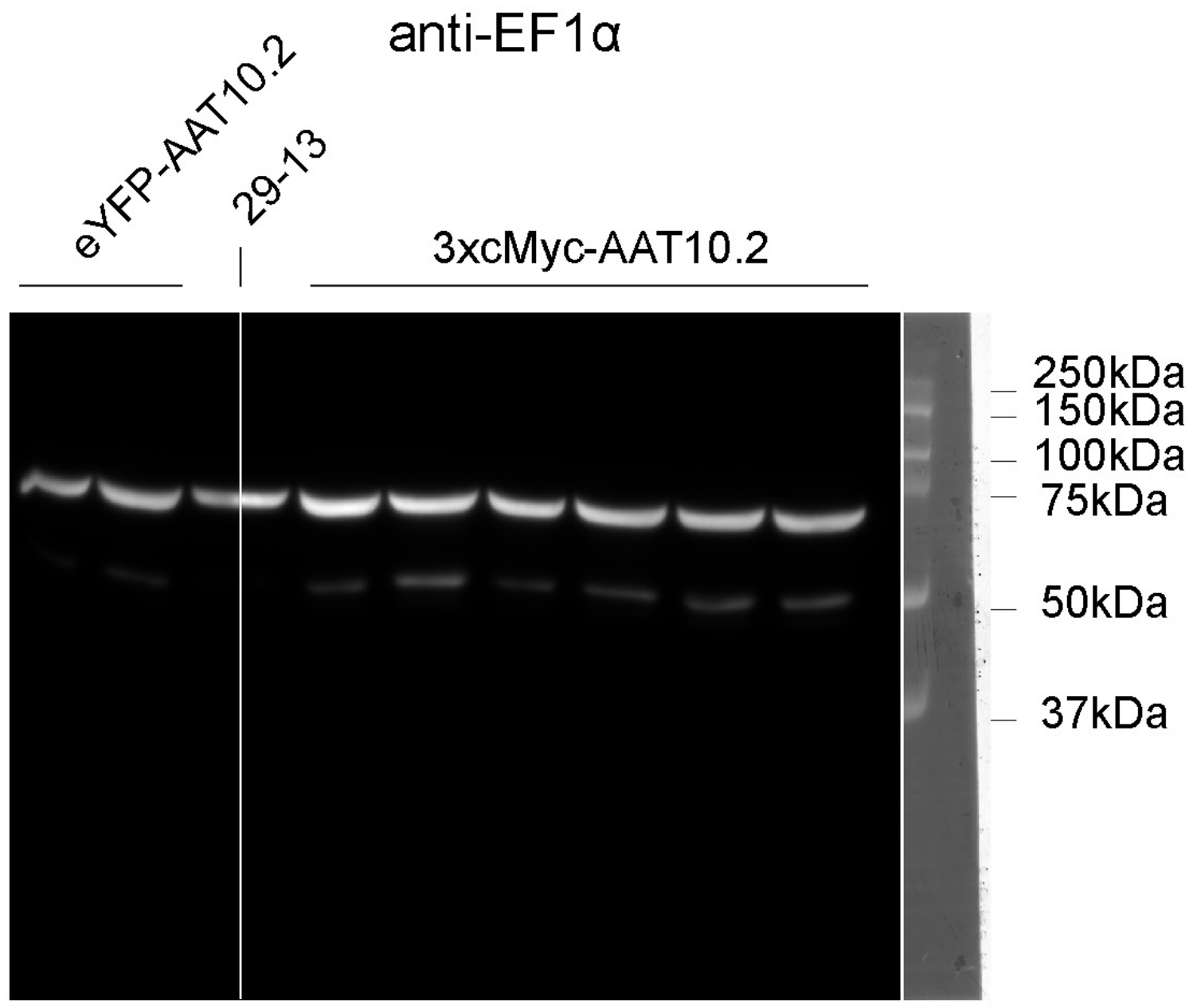

Suppl. Figure 17. Unedited full blots of Suppl. Figure 8A and 8B.

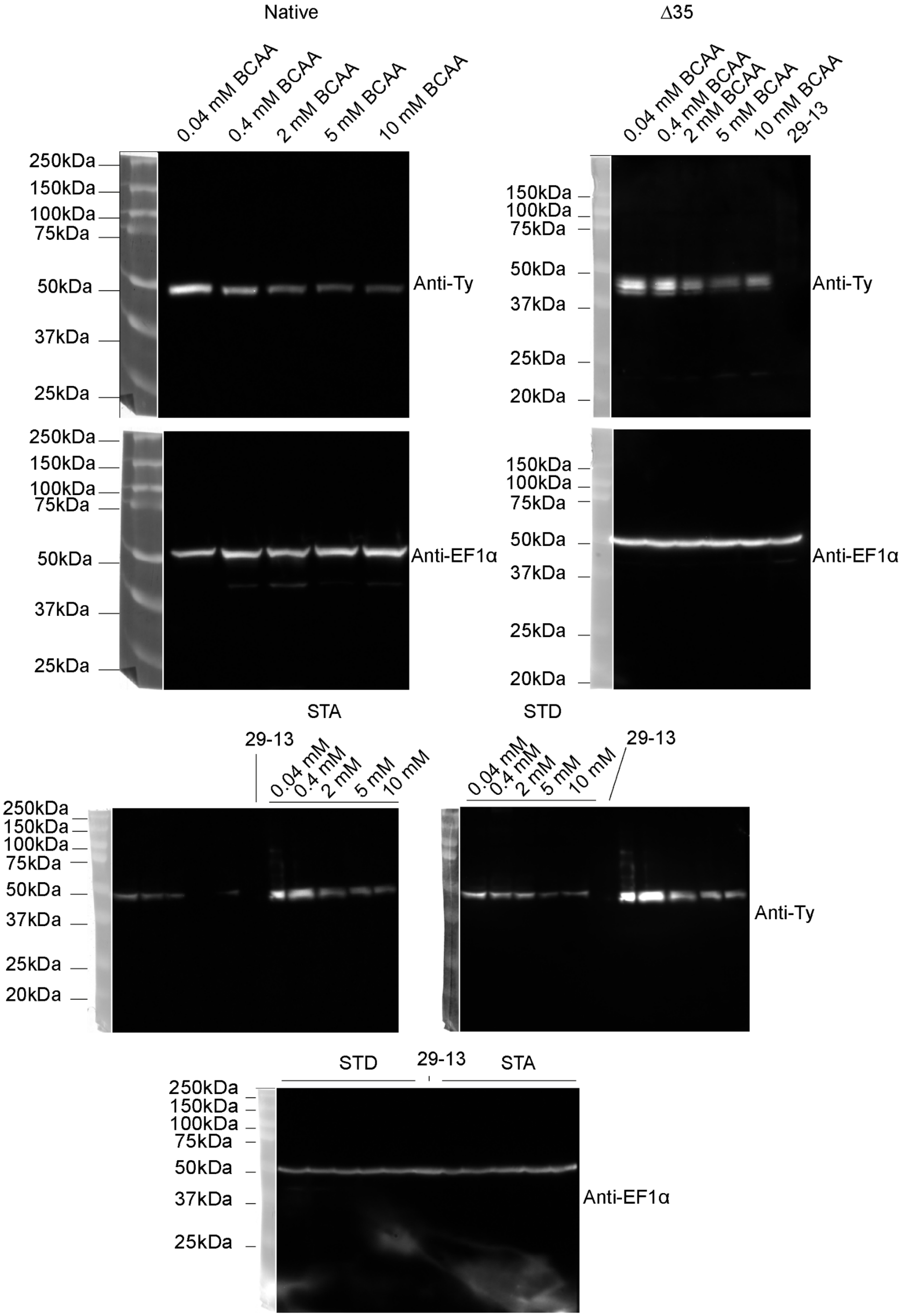

Suppl. Figure 18. Unedited full blots of Suppl. Figure 10A.

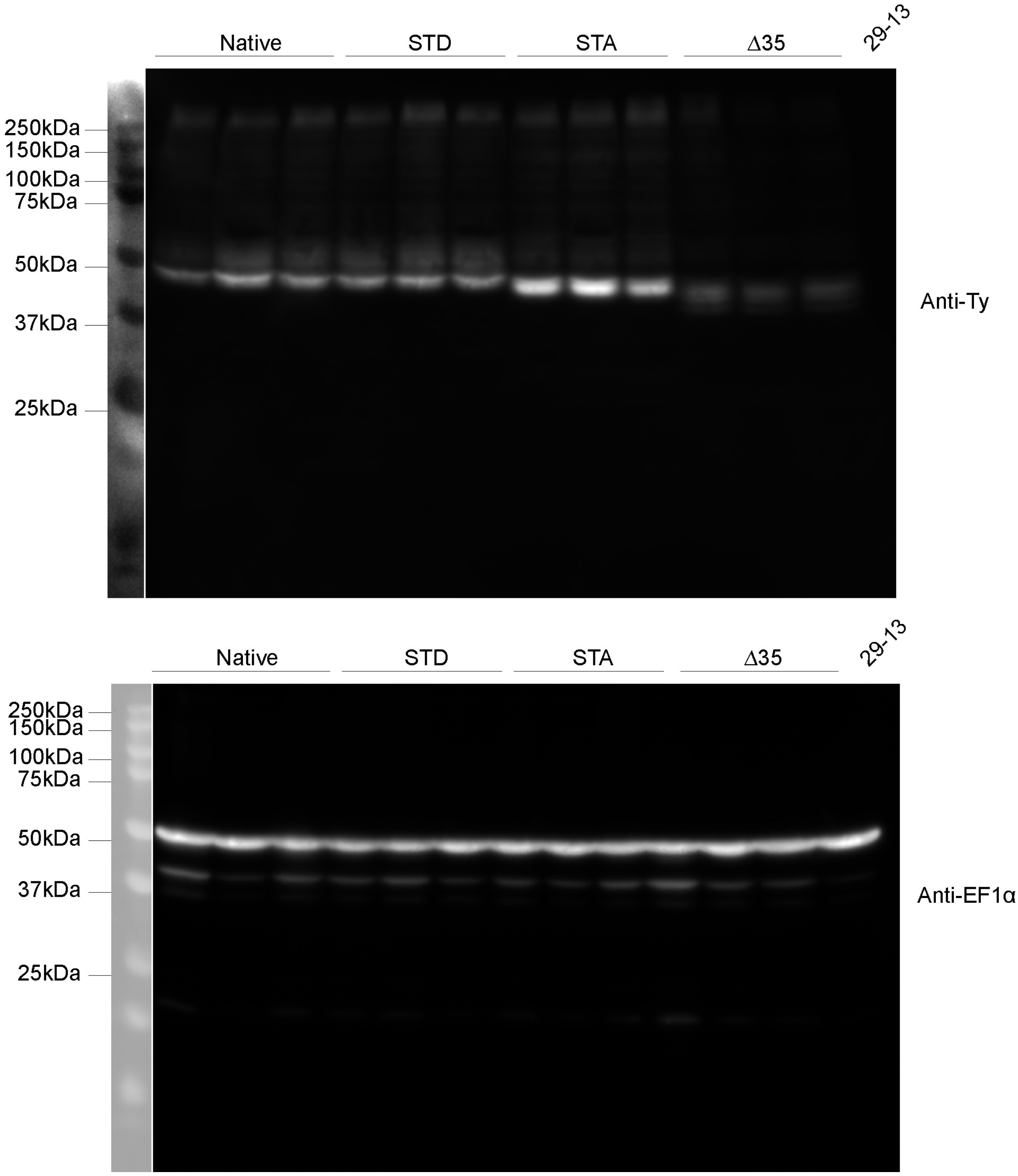

Suppl. Figure 19. Unedited full blots of Supplementary Figure 10C.
